# Effectiveness of the PPR Sungri-96 Vaccine in Sheep and Goats in India: A Nationwide Post-Vaccination Seromonitoring Study Towards PPR Elimination

**DOI:** 10.3390/v18101102

**Published:** 2026-10-06

**Authors:** Vinayagamurthy Balamurugan, Kirubakaran Vinod Kumar, Rakshit Ojha, Ponnidurai Malini, Ramachandra Deshpande, Prajakta Prashant Bokade, Annett Helcita Dsouza, Shweta Priya, Anand Asha, Archana Pal, Gangarajareddy Deekshitha, Mahadevappa Swathi, Shakuntala Krishnaiah Harshitha, Shanmugam Chandra Sekar, Kuralayanapalya Puttahonnappa Suresh, GurrappaNaidu Govindaraj, Divakar Hemadri, Nayakvadi Shivasharanappa, Sujit Nayak, Njeumi Felix, Baldev Raj Gulati, Satya Parida

**Affiliations:** 1 Indian Council of Agricultural Research—National Institute of Veterinary Epidemiology and Disease Informatics (ICAR-NIVEDI), Yelahanka, Bengaluru 560119, Karnataka, India; vinodkmr33@gmail.com (K.V.K.); ricky.ojha@gmail.com (R.O.); maliniponnidurai@gmail.com (P.M.); dramchandra643@gmail.com (R.D.); prajb95@gmail.com (P.P.B.); annettdsouza4099@gmail.com (A.H.D.); shweta2000jha@gmail.com (S.P.); ashareddy457@gmail.com (A.A.); archanapal307@gmail.com (A.P.); deekshitha0722@gmail.com (G.D.); swathi.par10@gmail.com (M.S.); harshi79sk@gmail.com (S.K.H.); suresh.kp@icar.org.in (K.P.S.); govindaraj.naidu@icar.gov.in (G.G.); divakar.hemadri@icar.org.in (D.H.); shivasharanappa.n@icar.org.in (N.S.); baldev.gulati@icar.org.in (B.R.G.); 2 Indian Council of Agricultural Research— Indian Veterinary Research Institute (IVRI), Mukteswar, Nainital 263138, Uttarakhand, India; schand_vet@yahoo.co.in; 3 Department of Animal Husbandry & Dairying (DAHD), Krishi Bhawan, New Delhi 110001, India; sujit.nayak@nic.in; 4 Food and Agriculture Organization of United Nations (FAO), Viale delle Terme di Caracalla, 00153 Rome, Italy; felix.njeumi@fao.org (N.F.); satya.parida@fao.org (S.P.)

**Keywords:** PPR eradication, epidemiological units, post-vaccination seromonitoring, vaccine effectiveness, India

## Abstract

India launched a nationwide Peste des Petits Ruminants (PPR) Eradication Programme in 2023 with the objective of achieving countrywide control and elimination of PPR by 2030, adapting a systematic mass vaccination, surveillance, and seromonitoring strategy in alignment with the WOAH/FAO Global Control and Eradication Strategy (GCES) under the PPR Global Eradication Programme (PPR-GEP) 2030. As part of the first round of mass vaccination, the present study evaluates the effectiveness of the PPR Sungri-96 (Lineage IV) vaccine in sheep and goats in fields across all the states in India, with particular emphasis on the post-vaccination evaluation (PVE) as per WOAH guidelines. A total of 24,542 post-vaccination (30–90 days) serum samples were collected from sheep and goats aged 6–12 months from 2528 epidemiological units (epi-units, villages) across 24 states of India, following GCES guidelines. Sampling was conducted according to state vaccination schedules during January 2023–December 2024, ensuring appropriate post-vaccination immune-response windows. Serum samples were analysed to detect PPR virus (PPRV)-specific antibodies using the native PPR competitive ELISA (c-ELISA) kit. The study demonstrated an overall post-vaccination seroconversion rate of 69.3% (95% CI: 68.7–69.8%) among small ruminants, indicating a substantial vaccine-induced immune response under field conditions in the target age group with desirable vaccine effectiveness. Exceptionally high post-vaccination seroconversion was observed in Telangana (91.6%) and Andhra Pradesh (82.3%), reflecting effective implementation of vaccination campaigns in these two states. Overall, 61% (1553/2528) of the evaluated epi-units recorded 70% seroconversion post vaccination, whereas 22% (510/2287) of epi-units showed ≥70% seroprevalence pre vaccination in sheep and goats aged 6 to 12 months. These findings demonstrate the effectiveness of the PPR Sungri-96 vaccine in inducing protective immunity under field conditions in accordance with GCES guidelines. The observed inter-state variations in immune response may be attributed to differences in vaccination coverage, implementation efficiency, animal demographics, husbandry practices, and local epidemiological conditions. However, some states failed to achieve the desired level of post-vaccination immunity, indicating the need for intensified vaccination efforts and improved campaign implementation in such regions. Continued mass vaccination, strengthened surveillance, real-time disease monitoring, farmer awareness programs, and capacity-building initiatives for veterinary services are critical for improving vaccine effectiveness and accelerating progress towards a PPR-free India. This study provides important field evidence supporting the effectiveness of the PPR Sungri-96 vaccine and contributes significantly to both national and global PPR eradication initiatives.

## 1. Introduction

Peste des Petits Ruminants (PPR), commonly known as small ruminant plague, is a highly contagious viral disease that primarily affects sheep and goats, with occasional spillover infections reported in wild ruminants. The disease is characterized by fever, ocular and nasal discharge, erosive stomatitis, enteritis, diarrhea, bronchopneumonia, and high morbidity and mortality, leading to substantial socio-economic losses among smallholder livestock farmers in endemic regions [1]. PPR is caused by the PPR virus (PPRV), a member of the genus *Morbillivirus* (species *Morbillivirus caprinae*) in the family *Paramyxoviridae*, and is recognized by the World Organisation for Animal Health (WOAH) as a major transboundary animal disease (TAD) of global importance [1,2]. The disease remains endemic across large parts of Africa, the Middle East, and Asia, while recent incursions into Europe, including Bulgaria, Greece, and Romania in 2024 and Hungary, Albania, Kosovo, and Croatia in 2025, have highlighted the continuing threat of transboundary spread [3,4]. PPRV Lineage IV was also reported for the first time in Vietnam in October 2025 [5]. Considering the significant economic impact and transboundary nature of the disease, WOAH and FAO launched the Global Control and Eradication Strategy (GCES) and the PPR Global Eradication Programme (PPR-GEP), aiming for global eradication of PPR by 2030 following the successful eradication model of rinderpest [6,7,8,9,10,11]. Vaccination remains the cornerstone for effective prevention and control of PPR. Currently, two live attenuated vaccines, the Nigeria 75/1 and Sungri-96 strains, are widely used globally and provide long-lasting protective immunity against circulating PPRV strains [6,7,8,9,10,11]. These vaccines are recognized in the WOAH/FAO eradication framework and are critical tools for achieving interruption of virus transmission and establishment of herd immunity.

India possesses one of the world’s largest small ruminant populations and has experienced endemic circulation of PPR for several decades. To reduce the disease burden, the Government of India (GoI) initiated focused vaccination campaigns during outbreaks from 2006 onwards, followed by implementation of the PPR-Control Programme in 2011 through the Department of Animal Husbandry and Dairying (DAHD). The program emphasized strategic mass vaccination, rapid diagnosis, surveillance, outbreak monitoring, and disease prevention [6,7,8,9,10,11]. These sustained vaccination efforts resulted in a progressive decline in PPR outbreaks and significant changes in disease occurrence, severity, and geographical distribution across the country. In alignment with the WOAH/FAO GCES framework, India launched the PPR Eradication Programme (PPR-EP) during 2022–2023 under the Critical Animal Disease Control Programme (CADCP) of the Livestock Health and Disease Control Programme (LHDCP) scheme of DAHD, GoI. The national strategic plan aims to achieve comprehensive vaccination of sheep and goats, interruption of virus transmission by 2028, and official freedom from PPRV infection by 2030 [6,7,8,9,10,11]. The program includes repeated rounds of mass vaccination targeting all eligible sheep and goats above 4 months of age, including animals in migratory, remote, and border regions, with the objective of achieving >90% vaccination coverage and sustaining herd immunity above the threshold required for interruption of transmission. The strategy also emphasizes post-vaccination seromonitoring, outbreak investigations, epidemiological surveillance, and real-time disease monitoring.

Despite substantial progress, sporadic outbreaks continue to occur in some regions of India, largely due to gaps in vaccination coverage, animal movement, population turnover, and surveillance bottlenecks [9]. Strengthening epidemiological surveillance and assessing post-vaccination seromonitoring immunity therefore remain essential components of the national eradication program. In this context, ICAR-NIVEDI, with support from DAHD, GoI, initiated a nationwide seromonitoring and surveillance program following WOAH/FAO GCES guidelines. Currently, the Sungri-96 strain Lineage IV live attenuated vaccine is the only commercially used PPR vaccine in India and has been shown to induce long-term protective immunity against circulating PPRV strains [10,11]. Evaluation of vaccine effectiveness under real-world field conditions is essential for understanding the level of seroconversion and immunity achieved following mass vaccination campaigns under the PPR-EP. Achieving and sustaining herd immunity above 70–80% through high vaccination coverage and effective vaccine performance under field conditions is critical for the interruption of virus transmission and successful eradication of PPR [11].

Therefore, the present study evaluated the effectiveness of the PPR Sungri-96 (Lineage IV) vaccine in sheep and goats under real-world field conditions across India through post-vaccination seromonitoring following the first round of mass vaccination implemented under the PPR Eradication Programme. The study aimed to assess vaccine-induced seroconversion in accordance with WOAH/FAO GCES guidelines under the PPR-GEP 2030 framework, thereby generating evidence to support strategic vaccination and eradication efforts in India.

## 2. Materials and Methods

### 2.1. Study Area

This study was conducted across Indian states representing diverse agro-climatic zones and small ruminant production systems (Table S1) [12]. The implementation of the PPR Control Programme (PPR-CP) in India followed a phased approach. The first phase (2010–2011) included southern and western states such as Maharashtra, Andhra Pradesh, Telangana, Karnataka, Keralam, and Tamil Nadu. The second phase, initiated in 2014, expanded coverage to the remaining states of India. Under the National Strategic Plan (NSP) for PPR eradication, all participating states completed at least one round of mass vaccination during 2023 and 2024. The states/Union Territories included Andhra Pradesh, Assam, Bihar, Chhattisgarh, Gujarat, Haryana, Himachal Pradesh, Jharkhand, Karnataka, Keralam, Madhya Pradesh, Maharashtra, Manipur, Meghalaya, Mizoram, Odisha, Puducherry, Punjab, Rajasthan, Sikkim, Tamil Nadu, Telangana, Uttar Pradesh, and West Bengal. These regions encompass a variety of geographic landscapes ranging from coastal plains and tropical zones to arid regions, plateaus, and mountainous terrains, thereby reflecting heterogeneity in livestock density, management systems, and husbandry practices [9,11]. Further, these states were included due to the availability of complete data on vaccine batch details, manufacturer, and cold-chain maintenance, along with the completion of first-round mass vaccination under NSP for PPR eradication and the implementation of GCES-compliant seromonitoring. All serum samples were collected within 30–90 days post-vaccination evaluation (PVE), ensuring a uniform post-vaccination assessment window for evaluating field seroconversion and vaccine effectiveness. The present study includes 24 states that completed vaccination activities and participated in systematic seromonitoring as per the GCES guidelines of WOAH/FAO [6,7,9]. According to the 20th Livestock Census of India (2019), the study states collectively contain approximately 70.76 million sheep and 145.17 million goats, representing a total small ruminant population of about 215.93 million animals (Table S1).

### 2.2. Study and Target Population

The study population comprised all susceptible sheep and goats within the selected states. Villages were considered epidemiological units (epi-units) due to their distinct socio-economic, ecological, and animal husbandry characteristics. The target group for vaccine seroconversion assessment included small ruminants aged 6–12 months, selected as the primary sentinel age cohort for evaluating field immune response following vaccination. Since the PPR Sungri-96 strain vaccine is non-DIVA (Differentiating Infected from Vaccinated Animals), serological differentiation between infection- and vaccine-induced antibodies was not possible; hence, this age group naïve population-level seroconversion was used as the primary indicator of immunological response.

### 2.3. Study Design and Sampling Strategy

A cross-sectional study design was employed to assess post-vaccination seroconversion in small ruminant populations following the first round of mass vaccination under the PPR-EP during 2023 and 2024. Sampling followed a stratified, two-stage cluster sampling design based on WOAH/FAO GCES Protocol 1 guidelines (Table S2) [6,7]. In the first stage, epi-units (villages) were selected as clusters from each state. In the second stage, animals within selected epi-units were sampled. A comprehensive sampling frame was developed using village-wise livestock population data from the 20th Livestock Census (2019). Epi-units were selected through randomization, with inclusion criteria requiring a minimum population of ≥500 or 100 sheep and goats per unit, depending on the population size in different states. Sampling was supported using the epi-calculator software V1 developed by the ICAR-National Institute of Veterinary Epidemiology and Disease Informatics (NIVEDI), Bengaluru. Within each epi-unit, 3–4 households or flocks were randomly selected, and a maximum of 10 animals belonging to the 6–12 month age group were included for serological assessment per epi-unit [13].

### 2.4. Sample Size Estimation

Sample size was determined according to WOAH/FAO GCES guidelines [6,7] using the formula *n* = (Z^2^ × P (1 − P))/e^2^, where *n* is the sample size for the state; Z is the value from the standard normal distribution corresponding to the 95% confidence level (1.96); P is the prevalence, which is to be taken as 30% at field level; and ‘e’ is the precision of the sample size estimate and is normally set at 10% (0.10), as is the acceptable sampling error. To account for the diagnostic sensitivity and specificity of the PPR competitive ELISA (c-ELISA) [10,14], sample size adjustments were applied as per standard epidemiological methods. A two-stage cluster sampling framework was used, with 108–120 epi-units selected per state, exceeding the minimum requirement of 97 units recommended under GCES guidelines [11]. This ensured adequate statistical power and representativeness across heterogeneous livestock populations. Within each epi-unit, secondary sampling units (animals) were determined using hypergeometric distribution assumptions. A fixed cluster size of 9–10 animals per epi-unit was adopted, yielding a minimum sample size of approximately 1080 animal samples per state (108 epi-units × 10 animals).

### 2.5. Sample and Data Collection

**Figure 1:**
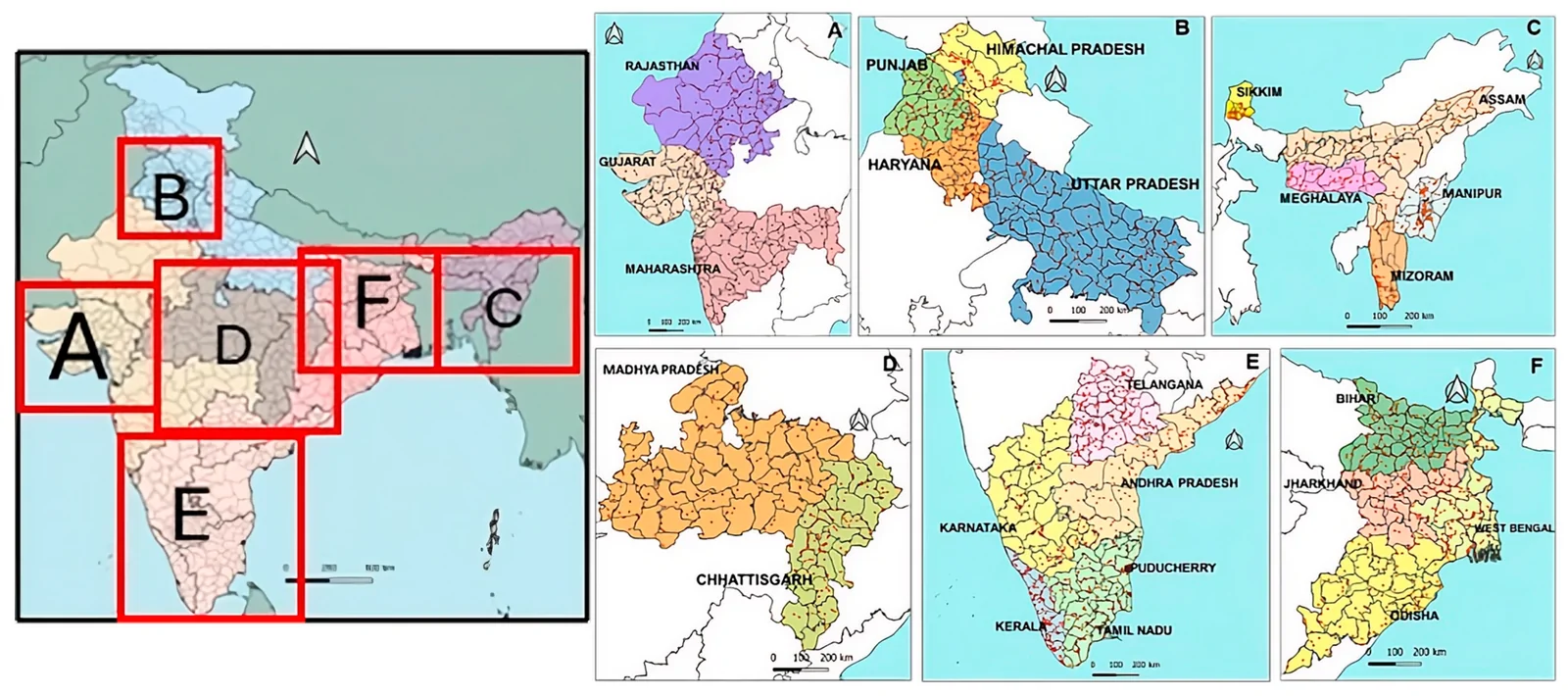
Locations of surveyed epi-units (villages) across 24 Indian states and union territories in different zones are depicted as dots in GIS-based maps of India. Red boxes in India map in each panel indicate the geographical position of each state in different zones. Panel (**A**): Western Zone (Gujarat, Rajasthan, and Maharashtra). Panel (**B**): Northern Zone (Punjab, Haryana, Himachal Pradesh, and Uttar Pradesh). Panel (**C**): Northeastern Zone (Mizoram, Manipur, Meghalaya, Assam, and Sikkim). Panel (**D**): Central Zone (Chhattisgarh and Madhya Pradesh). Panel (**E**): Southern Zone (Andhra Pradesh, Telangana, Karnataka, Tamil Nadu, Keralam, and Puducherry). Panel (**F**): Eastern Zone (Odisha, Bihar, Jharkhand, and West Bengal). These maps present the spatial distribution of surveyed epi-units sampled for immune response study after first round of mass vaccination conducted under the National PPR Eradication Programme during 2023 and 2024.

Post-vaccination seromonitoring was conducted exclusively in 6–12-month-old sheep and goats across selected states, approximately 30–90 days following mass vaccination campaigns under the PPR-EP. Sampling timelines varied by state based on vaccination schedules in 2023 and 2024, ensuring appropriate post-vaccination immune response windows. Across all states, structured field investigations were conducted between January 2023 and December 2024. In total, 2528 epi-units were surveyed across 24 states. From each epi-unit, up to 10 serum samples were collected from randomly selected sheep and goats. In mixed flocks, species-specific sampling was ensured; where only sheep or goats were present, samples were collected from the available species. In addition to biological sampling, structured questionnaires were used to collect data on host factors including species, breed, sex, and management practices [7]. The geographic distribution of epi-units surveyed across states in different zones is illustrated in the figure below, which highlights nationwide coverage of the seromonitoring framework, and were recorded and mapped using QGIS software (version 3.30.0) (Figure 1 and Figure S1).

### 2.6. Serological Testing and Data Analysis

Serum samples were tested using PPR competitive ELISA (c-ELISA) kit (IDvet, Grabel, France) for the detection of antibodies against the hemagglutinin (H) protein of PPR virus. Samples with percentage inhibition (PI) ≥ 40% (for having high sensitivity) were considered positive for PPRV-specific antibodies, following validated diagnostic cut-offs for field serosurveillance [10,14]. The seroconversion was calculated by dividing the number of positive animals by the total number of animals tested. Associations between seropositivity and host factors (species, age, breed, and sex) were evaluated using Pearson’s Chi-square test and Fisher’s exact test where appropriate. Point estimates of seroconversion at the national, state and host-factor level are additionally presented with 95% confidence intervals (Wilson score method), reported in Table 1 and Table 2. Given the nested, two-stage cluster sampling design (animals within epi-units within districts, states and zones), these Chi-square/Fisher’s tests were interpreted primarily to identify broad patterns rather than as fully design-adjusted inferential estimates; this is discussed further as a limitation in Section 4. Statistical analyses were performed using Microsoft Excel (2016) and standard epidemiological procedures [15]. Available laboratory data (unpublished) from pre-vaccination serosurveys (6–12-month-old animals) conducted under the PPR-EP were utilized in the present seromonitoring study to assess protective epi-units. An increase in the proportion of epi-units achieving high protection levels (≥70% seropositivity) in more than 50% of the surveyed epi-units, as per the sampling design, indicates successful vaccine-induced seroconversion and field effectiveness in accordance with GCES guidelines of the PPR-GEP [7]. This pre-vaccination dataset served as a critical baseline for assessing immunity after post-vaccination in the 6–12-month age cohort under the national program.

### 2.7. Vaccine Effectiveness Assessment 

The PPR Sungri-96 strain (Lineage IV) vaccine is nationally deployed under the PPR-EP in India. The vaccine is produced by multiple authorized manufacturers including state biological institutes such as the Telangana State Veterinary Biological & Research Institute (TSVBRI) and the Institute of Animal Health & Veterinary Biologicals (IAH&VB), Karnataka, and the Haryana Veterinary Vaccine Institute (HVVI), Hisar, Haryana, as well as multinational private-sector manufacturers such as M/s Hester Biosciences Limited under centralized procurement by the DAHD, GoI. The Sungri-96 vaccine technology of the ICAR-Indian Veterinary Research Institute (IVRI) has been transferred to several public and private vaccine manufacturers to support large-scale production and national vaccination campaigns. In the present study, more than 50 vaccine batches sourced from different authorized manufacturers were utilized under field conditions across multiple states. All vaccine batches underwent stringent quality-control procedures prior to field deployment, including batch-wise potency testing, third-party quality verification, and centralized release protocols in accordance with national regulatory and procurement guidelines. These measures ensured uniform vaccine quality, potency, safety, and compliance with prescribed standards before administration in the field. Monitoring vaccination programs is crucial, and an increase in the proportion of epi-units (more than 50% of the studied epi-units, as per the sampling plan) with high protection levels (≥70%) indicates successful seroconversion, as per the GCES guidelines of the PPR-GEP [6,7]. Vaccine efficacy was defined as the proportion of vaccinated young animals demonstrating seroconversion 30–90 days post vaccination, while effectiveness evaluates how well the program protects populations in the field, considering storage, distribution, and coverage under field conditions. Vaccine effectiveness was calculated by multiplying vaccination coverage by seroconversion [6,7]. An increase in the proportion of epi-units achieving ≥70% immunity was interpreted as evidence of successful immunization and progression toward herd immunity thresholds required for PPR elimination.

## 3. Results

### 3.1. National Level

Post-vaccination seromonitoring was conducted across 24 states of India in small ruminants aged 6–12 months following the first round of mass vaccination under the PPR-EP. The study covered 565 districts, 1675 taluks, 2528 epi-units, and 24,542 serum samples, providing extensive representation across diverse agro-climatic zones and livestock production systems in the country (Table 1). The evaluation included multiple vaccine manufacturers and more than 50 vaccine batches, enabling assessment of the field performance and consistency of the PPR Sungri-96 strain Lineage IV vaccine under real-world conditions. The overall seroconversion rate for PPRV antibodies was 69.3% (95% CI: 69.0–70.0). States such as Telangana (91.6%) and Andhra Pradesh (82.3%) demonstrated consistently high seroconversion, indicating strong field-level vaccine performance and coverage. In contrast, states such as Meghalaya (53.1%) and Himachal Pradesh (53.9%) exhibited comparatively lower immunity levels, suggesting the need for intensified vaccination coverage and follow-up campaigns. Species-wise analysis revealed higher seroconversion in sheep (75.6%) compared to goats (67.4%) following vaccination. The results of an evaluation of PPR Sungri-96 vaccine-induced seroconversion or PPRV antibody immune response in small ruminants (6–12 Months) at the epidemiological unit level across different states of India are presented in Table 1. Significant differences in seropositivity were observed between species across states (χ^2^ = 1093.5, *p* < 0.005). Similarly, significant variation was recorded among states for both sheep (χ^2^ = 487.9, *p* < 0.005) and goats (χ^2^ = 613.2, *p* < 0.005), indicating marked regional heterogeneity in immune response. Host factors including sex (χ^2^ = 28.22, *p* < 0.05), species (χ^2^ = 139.92, *p* < 0.05), and management practices (χ^2^ = 45.84, *p* < 0.05) were significantly associated with seroconversion, whereas breed showed no significant association (χ^2^ = 1.22, *p* > 0.05). Chi-square analysis results of PPR vaccine antibody immune response in small ruminants are presented in Table 2.

### 3.2. Epidemiological Unit Level

**Figure 2:**
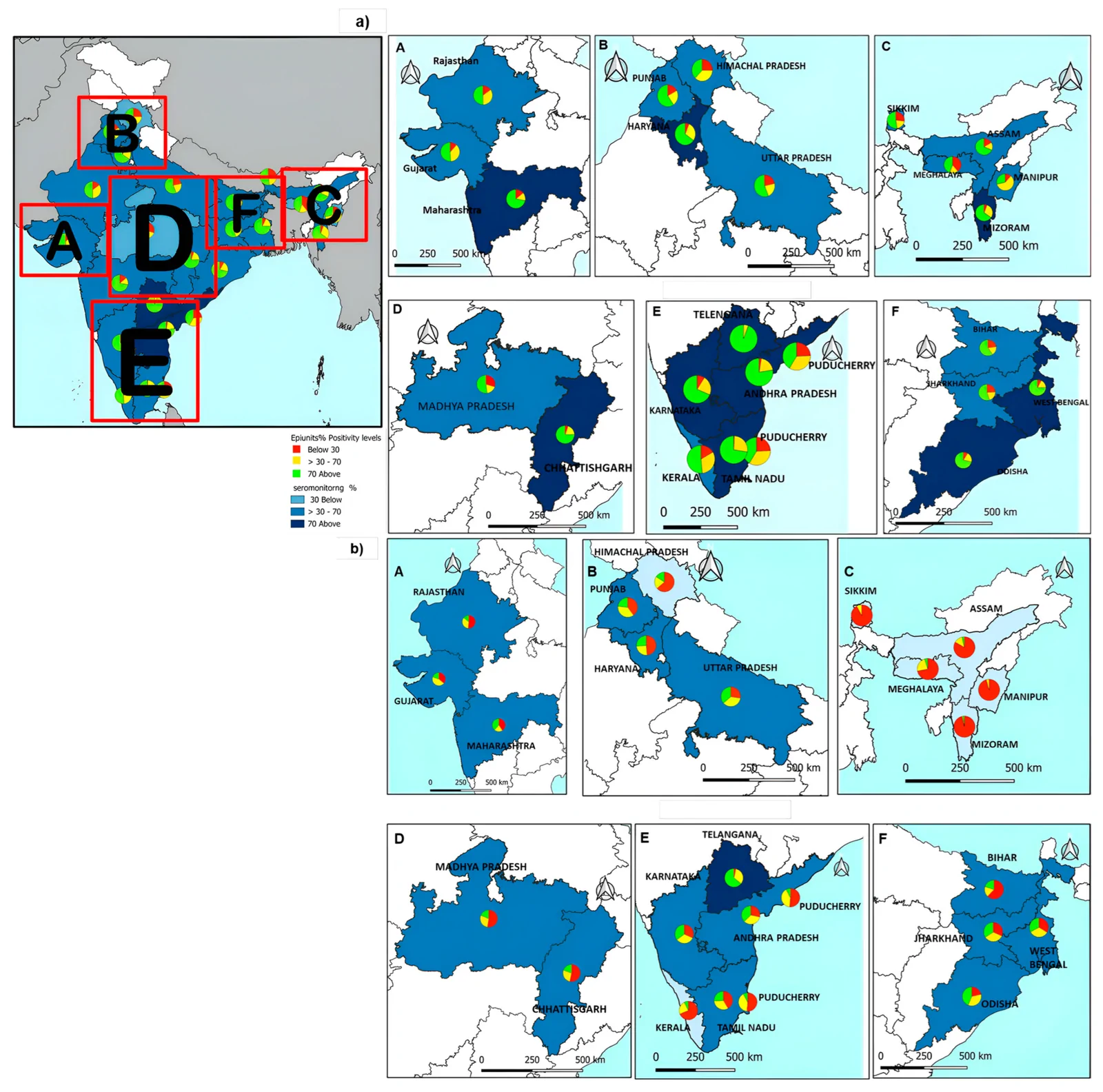
(**a**) State-level immune response to PPR vaccine (% seroconversion) in small ruminants across 24 States/UTs of India. Shaded gradients represent state-level immune response (%) based on seroconversion, while pie charts illustrate the distribution of epi-units by seropositivity levels: red for below 30%, yellow for 30–70%, and green for above 70%. The map depicts the PPR vaccine effectiveness in goats and sheep in india after the first annual mass vaccination implemented under the National PPR Eradication Programme of the Livestock Health and Disease Control Programme (LHDCP), Government of India. (**b**) Similarly, state-wise PPRV antibody seroprevalence in small ruminants was assessed during the baseline serosurvey before vaccination. The data generated were used to evaluate the achievement of ≥70% protection in over 50% of the surveyed epi-units, indicating successful vaccine-induced seroconversion and field effectiveness as per the PPR-GEP GCES guidelines. Red boxes in India map in each panel (**A**–**F**) indicate the geographical position of each state in different zones of India as stated in the Figure 1.

At the epi-unit level, 61% (*n* = 1553) of units achieved ≥70% seroconversion, indicating strong vaccine-induced herd immunity in a majority of locations. However, 24.3% (*n* = 614) of epi-units showed moderate seroconversion (31–70%), and 14.3% (*n* = 361) remained below 30%, reflecting residual immunity gaps in certain pockets (Figure 2). Overall, post-vaccination seromonitoring of PPR vaccine antibodies immune response in small ruminants, with the distribution of epidemiological units, is summarized in Table 3 and Table S3. Details of the post-vaccination PPRV antibody seroconversion status in small ruminants in different epi-units of various districts of studied 24 states/UTs of India provided in the Supplementary Tables S4–S27 and Figures S2 and S3:

The Southern Zone (650 epi-units) exhibited the highest overall immune response. Telangana recorded the highest seroconversion (91.6%, 95% CI: 90–93), followed by Andhra Pradesh (82.3%), Tamil Nadu (77.6%), Karnataka (73.4%), Keralam (61.9%), and Puducherry (55.6%). Telangana had 94.2% of epi-units achieving ≥ 70% seroconversion, with no units below 30%. Andhra Pradesh, Tamil Nadu, and Karnataka also demonstrated high proportions of protected epi-units, while Keralam and Puducherry showed relatively lower coverage, with a higher proportion of units falling below the protective threshold. The Central Zone (226 epi-units) showed moderate seroconversion levels. Chhattisgarh recorded significantly higher immunity (78.8%, 95% CI: 76–81) compared to Madhya Pradesh (58.3%, 95% CI: 56–60). Correspondingly, 75.5% of epi-units in Chhattisgarh achieved ≥70% seroconversion, whereas Madhya Pradesh showed only 51.9% of epi-units above this threshold.

The Western Zone (335 epi-units) demonstrated an overall seroconversion of 69.7% (95% CI: 68–72). Maharashtra showed the highest response (75.2%), followed by Rajasthan (64.7%) and Gujarat (64.5%). Maharashtra had 71.9% of epi-units with ≥70% seroconversion, while Gujarat and Rajasthan showed moderate immunity levels with a higher proportion of intermediate- and low-response units. The Northern Zone (430 epi-units) achieved an overall seroconversion of 65.1% (95% CI: 64–66). Haryana showed the highest seroconversion (72.8%), followed by Punjab (67.8%), Uttar Pradesh (63.3%), and Himachal Pradesh (53.9%). In Haryana, 65.2% of epi-units achieved ≥70% seroconversion, whereas Himachal Pradesh had the lowest proportion of high-immunity units and the highest proportion of low-seroconversion epi-units.

In the Eastern Zone (437 epi-units), West Bengal showed the highest seroconversion (79.7%, 95% CI: 77–82), followed by Odisha (76.9%), Bihar (64.2%), and Jharkhand (61.4%). West Bengal and Odisha had >70% seroconversion in 67.5% and 71.4% of epi-units, respectively. In contrast, Bihar and Jharkhand showed a higher proportion of low-seroconversion epi-units, indicating heterogeneous vaccine response across the region. The Northeastern Zone (450 epi-units) showed an overall seroconversion of 63.1% (95% CI: 62–65). Mizoram recorded the highest immunity (74.2%), followed by Assam (69.7%), Sikkim (59.7%), Manipur (57.8%), and Meghalaya (53.1%). Mizoram and Assam demonstrated relatively strong vaccine responses, whereas Meghalaya and Manipur showed lower seroconversion and a higher proportion of epi-units below the protective thresholds.

### 3.3. Vaccine Effectiveness in the Fields

Overall, 61% of epi-units achieved ≥70% seroconversion, indicating successful field-level vaccine performance in most surveyed regions. The observed seroconversion pattern corresponds to an estimated vaccine effectiveness of close to 70% under real-world field conditions. However, variability across regions indicates that while vaccine performance is robust at the national level, field effectiveness is influenced by vaccination coverage, cold-chain maintenance, and implementation efficiency. The combined analysis of vaccination coverage and seroconversion further supports strong overall vaccine effectiveness under programmatic conditions while also identifying specific geographic areas requiring intensified immunization strategies. State-level seroconversion and effectiveness estimates are summarized in Table 1.

## 4. Discussion

Generally, vaccine efficacy refers to the protective effect of a vaccine under controlled experimental conditions, whereas vaccine effectiveness reflects its performance under routine field conditions in heterogeneous populations influenced by animal, management, and environmental factors. Vaccine effectiveness is influenced by factors such as vaccine handling and administration, host characteristics (species, breed, age, and physiological status), pathogen dynamics, vaccination coverage and schedules, population immunity, and prevailing environmental and management conditions. Achieving and sustaining herd immunity above the critical threshold of 70–80% is essential for interrupting PPRV transmission and progressing toward global eradication under the PPR-GEP 2030. In this context, the present study provides large-scale field evidence on the effectiveness of the PPR Sungri-96 (Lineage IV) vaccine under routine PPR-EP programmatic conditions across diverse geographical regions, production systems, and sheep and goat populations in India based on post-vaccination seromonitoring assessment. This nationwide assessment, conducted across 24 states representing diverse agro-climatic zones, demonstrates that mass vaccination under the national strategic plan (NSP) has resulted in a substantial level of immune response. The observed overall seroconversion rate of 69.3% indicates that the vaccination program has achieved vaccine effectiveness levels of nearly 70% at the national scale under field conditions, despite inherent operational and ecological heterogeneity. The significantly higher seroconversion observed in sheep compared to goats is consistent with previous reports and may be attributed to differences in management systems, lifespan, and exposure risk. Sheep are often maintained under more stable production systems, whereas goats are frequently subjected to extensive grazing and higher movement/replacements of stocks, potentially affecting vaccine uptake and immune response consistency. Similar species-level differences have been reported in earlier PPR epidemiological studies and reinforce the need for species-specific targeting in vaccination strategies [16].

For comparative evaluation in the first round of the post-vaccination phase, the baseline serological findings were used to measure the impact of vaccination interventions implemented in young stock (6–12-month age group) with very little exposure to previous infections, particularly in epi-units where susceptibility was highest. The pre-vaccination serosurvey thus formed the reference framework against which seroconversion rates and immunity gains were assessed following vaccine deployment. Importantly, more than 60% of epi-units achieved ≥70% seroconversion, confirming strong programmatic performance of vaccine effectiveness in most regions. Importantly, the baseline analysis demonstrated that only a limited proportion of epi-units had achieved or exceeded the 70% seroprevalence threshold, which is generally considered indicative of the degree of protective herd immunity required to interrupt PPRV transmission. Overall, 61% of the evaluated epi-units recorded post-vaccination immune response levels above 70%, compared to only 22% of epi-units that showed ≥70% seroprevalence during the pre-vaccination serosurvey in the age group of 6 to 12 months. This contrast between pre- and post-vaccination immunity profiles enabled robust assessment of vaccine, effectiveness and population-level impact. The comparison further allowed for identification of epi-units that transitioned from low or moderate immunity (<70%) to protective immunity levels (>70%) following vaccination, thereby providing evidence for the success of targeted immunization strategies and supporting progress toward PPR eradication goals.

The zonal analysis highlights clear geographic variation in vaccine-induced immunity, reflecting differences in livestock density, vaccine coverage, management systems, implementation efficiency, and logistical constraints. Southern states such as Telangana, Andhra Pradesh, Tamil Nadu, and Karnataka consistently exhibited higher seroconversion levels, with several epi-units approaching or exceeding near-uniform immunity response, as reported earlier [13]. These results likely reflect earlier initiation of vaccination activities, better-established veterinary infrastructure, and improved campaign coverage in these regions. In contrast, comparatively lower seroconversion in parts of the Northeastern and Central zones suggests persistent operational challenges, including difficult terrain, smaller flock sizes, and logistical limitations affecting vaccination reach and post-vaccination monitoring [17]. A nationwide pre-vaccination cross-sectional serosurvey of 83,196 samples from 3099 epi-units across 33 States/UTs of India revealed an overall PPRV antibody seroprevalence of 40.53%, with substantial heterogeneity across states and epi-units (1.4–82.13%) (unpublished data). Notably, more than 1500 epi-units showed seroprevalence below 30%, reflecting pronounced immunity gaps and identifying regions at heightened risk of virus circulation and outbreak persistence, thereby underscoring the need for targeted mass vaccination interventions (Table 1). The dataset generated a representative snapshot of naturally acquired and previously vaccine-induced immunity levels across diverse agro-ecological and livestock production systems in the country.

The present findings also underscore the importance of epidemiological unit-level monitoring in evaluating vaccine performance. Although national-level seroconversion was high, a subset of epi-units (approximately 14.3%) showed seroprevalence of immune response below 30%, indicating localized immunity gaps. These pockets of low immunity are epidemiologically important, as they may sustain virus circulation and compromise elimination efforts if not addressed through targeted next annual mass vaccination campaigns. The observed heterogeneity across epi-units is consistent with the nature of large-scale mass vaccination programs, where variations in cold-chain integrity, field delivery efficiency, and small-ruminant population turnover can influence immune outcomes. Similar variability has been reported in other endemic settings, including Ethiopia, where post-vaccination seroconversion ranged widely depending on vaccine handling and field conditions [18], further supporting the role of implementation quality in determining vaccine effectiveness. Importantly, the study confirms that more than 50% of epi-units in all surveyed states achieved the GCES-recommended benchmark of ≥70% seroconversion, demonstrating compliance with WOAH/FAO standards for vaccine effective immunity. This provides strong evidence that the PPR Sungri-96 vaccine retains robust immunogenicity under field conditions and is capable of inducing protective immunity at scale when delivered through well-structured national programs.

In the northeastern states and certain hilly regions, variability was observed in the proportion of epi-units achieving ≥70% seropositivity between the pre- and post-vaccination periods (Table 1). In Assam, although only 3% of epi-units exhibited ≥70% seropositivity prior to vaccination, this proportion increased to more than 60% of epi-units following vaccination, likely reflecting high vaccination coverage and effective field-level implementation. In contrast, states such as Meghalaya and Manipur showed low baseline immunity, with <10% of epi-units achieving ≥70% seropositivity prior to vaccination, which improved to only about 40% of epi-units post-vaccination. A similar pattern was also observed in Himachal Pradesh in the northern region. These differences may be attributed to multiple factors, including variations in vaccination coverage and implementation efficiency, logistical constraints, maintenance of cold chain systems, accessibility challenges in remote and hilly terrains, animal husbandry practices, and the broader geographical and epidemiological heterogeneity influencing immune response across regions.

In general, achieving elimination requires not only high vaccine effectiveness at epi-units but also sustained vaccination coverage exceeding 90–95% and vaccine effectiveness > 70–80%, particularly in high-turnover small ruminant populations. The experience of countries such as Morocco highlights that maintaining high vaccination effectiveness at population level is critical for successful elimination of PPR [19]. In the Indian context, the current findings suggest that while overall vaccine performance is strong, targeted interventions are required in regions with lower seroconversion, particularly in parts of the Northeastern, Central, and select Western states. The use of a non-DIVA PPR c-ELISA system represents a methodological limitation, as it does not differentiate between vaccine-induced and infection-induced antibodies [14]. However, restricting sampling to the 6–12-month age group, which largely represents naïve or recently vaccinated animals, and conducting sampling within 90 days post-vaccination strengthens the inference that detected antibodies predominantly reflect vaccine-induced immune response or seroconversion. This approach aligns with WOAH/FAO GCES Protocol I recommendations for post-vaccination evaluation (PVE) in non-DIVA systems [6,7]. Furthermore, the sampled age cohort was largely too young to have completed a full disease season of natural field exposure in most of the surveyed regions, and reported PPR outbreak activity during the study period and areas was sparse, further supporting a predominantly vaccine-induced origin of the detected antibodies. Nonetheless, in the absence of a DIVA-compatible assay, a limited contribution from incidental natural exposure during the post-vaccination window cannot be entirely excluded, and this is acknowledged as a limitation of the present serological strategic approach. The young age group (6–12 months) was assumed to not have been exposed to PPR infection earlier.

A further limitation concerns the statistical treatment of the hierarchical, clustered sampling design. Individual animal units (*n* = 24,542) were nested within epi-units/villages (*n* = 2528), which were in turn nested within districts (*n* = 565), states (*n* = 24) and zones (*n* = 6). Because animals from the same village share common environmental exposure, management practices and vaccination-campaign implementation, observations within a cluster are not fully independent, and the unadjusted Pearson’s Chi-square and Fisher’s exact tests reported in Section 2.6 and Table 2 and Table 3 may therefore underestimate standard errors and inflate the nominal Type I error rate. To partially address this, the principal inferences of the present study are drawn at the epi-unit level (the proportion of epi-units achieving ≥70% seroconversion; Table 1 and Table 3) and at the state/zone level (Table 1), both of which are inherently less sensitive to within-cluster correlation than animal-level significance tests. We nonetheless agree that formal multilevel (mixed-effects) modeling, with village, district, state and zone fitted as nested random effects, would yield more statistically robust estimates of the host-factor associations reported here, and we have noted this as an explicit limitation of the present analysis and a priority for further analysis of the underlying dataset.

Sampling constraints in sparsely populated or geographically challenging regions, particularly in Northeastern states, also highlight the need for adaptive epidemiological frameworks. While the exclusion of very small epi-units was necessary for statistical robustness, it did not significantly bias overall estimates due to their inclusion in vaccination coverage. Nevertheless, future monitoring systems may benefit from flexible sampling designs tailored to region-specific livestock demographics. Overall, the findings demonstrate that the PPR Sungri-96 vaccine is highly effective under real-world field conditions, achieving substantial seroconversion and contributing meaningfully to vaccine immune response across India. The observed immunity levels support ongoing progress toward PPR elimination, while also identifying specific geographic pockets requiring intensified vaccination efforts and enhanced monitoring and surveillance. Strengthening cold-chain systems, improving last-mile delivery, and ensuring uniform vaccination coverage will be critical to closing existing immunity gaps [20,21].

In summary, this study provides strong field evidence supporting the effectiveness of the PPR Sungri-96 vaccine in achieving protective-level immunity under programmatic conditions. The results reinforce the feasibility of PPR elimination in India, provided that sustained high-coverage vaccination, targeted gap-filling strategies, and robust seromonitoring and surveillance systems are maintained in alignment with the PPR-GEP 2030 framework.

## 5. Statistical Interpretation and Program-Level Implications

The statistical analysis was descriptive and program-oriented, in line with the objective of assessing post-vaccination seromonitoring under the National PPR-EP, rather than identifying individual-level risk factors. Comparisons by species, sex, breed, and State/UT were therefore considered exploratory and intended primarily for program monitoring. The overall seroconversion of 69.3% and the increase in epi-units achieving the PPR GCES-recommended ≥70% seroprevalence threshold from 22% before vaccination to 61% after the first mass-vaccination round demonstrate a substantial improvement in population-level antibody status. These findings provide strong field evidence of the effectiveness of the Sungri-96 vaccine when deployed under national program conditions. However, because the pre- and post-vaccination surveys were independent cross-sectional assessments, the observed increase should not be interpreted as a formal individual-level vaccine-effectiveness estimate or direct measurement of protective immunity.

Considerable geographical variation was observed, with the proportion of epi-units meeting the ≥70% threshold ranging from 94.2% in Telangana to 44.3% in Meghalaya. This heterogeneity is more likely to reflect differences in vaccination coverage, campaign implementation, accessibility, cold-chain maintenance, timing, and population turnover than intrinsic differences in vaccine performance. These findings therefore highlight the need to prioritize subsequent vaccination rounds and program strengthening in areas with persistent immunity gaps. Finally, c-ELISA seroprevalence represents the proportion of animals with detectable PPRV antibodies and should be interpreted as an operational indicator of population-level immunity rather than direct proof of individual protective immunity. Overall, the findings demonstrate substantial progress following the first vaccination round while emphasizing the need for continued, targeted vaccination and improved program implementation to achieve uniformly high population immunity and support the national PPR eradication goal. The state- and zone-level heterogeneity observed nonetheless remains programmatically informative, pointing to implementation, coverage, and cold-chain factors—rather than the intrinsic performance of the vaccine—as the principal drivers of residual immunity gaps in specific geographies.

## 6. Limitations

Several limitations should be considered when interpreting these findings. First, the non-DIVA c-ELISA cannot distinguish vaccine-induced from infection-induced antibodies. Although sampling focused on a largely naïve young age group within 30–90 days after vaccination, a minor contribution from natural exposure cannot be completely excluded. In addition the seroprevalence sampling plan was adjusted for assay sensitivity and specificity and should therefore be regarded as an operational indicator of population antibody immunity status rather than a direct measure of individual protective immunity. Second, the multistage sampling design involved clustering of animals within epi-units, districts, States/UTs, and zones. Because individual-level management variables and consistent flock/household identifiers were not available, formal multilevel modeling and design-adjusted national estimates were not feasible. Exploratory associations were therefore assessed using Chi-square/Fisher’s exact tests, and the corresponding *p*-values should be interpreted cautiously. The convenience-based selection of animals within epi-units, as permitted under PPR GCES operational guidance, may also have introduced selection bias. Third, the study was based on a single post-vaccination cross-sectional assessment following the first annual vaccination round. The pre- and post-vaccination datasets comprised independent cross-sectional samples rather than longitudinal follow-up of the same animals; consequently, the observed increase in seroprevalence cannot be interpreted as a formal individual-level vaccine-effectiveness estimate or as evidence of the durability of immunity. In the absence of unvaccinated controls, causal inference regarding vaccine effectiveness is likewise limited. Fourth, vaccine batch and vaccination-coverage information were obtained largely from state-level program records, with batch information unavailable for some States/UTs, limiting detailed batch-specific assessment. Finally, very small epi-units were excluded in the sampling to improve analytical robustness, which may limit generalizability to sparsely populated or pastoral populations. These limitations notwithstanding, the large national sample, standardized laboratory procedures, and program-based sampling provide useful evidence for monitoring population-level immunity and guiding subsequent PPR vaccination strategies.

## 7. Conclusions

This study provides robust field evidence on the effectiveness of the PPR Sungri-96 (Lineage IV) vaccine under real-world conditions across 24 states of India, representing diverse agro-climatic zones and production systems, using vaccines from different manufacturers and more than 50 vaccine batches, thereby demonstrating the consistency and large-scale field performance of the vaccine under varied epidemiological and management conditions. The overall seroconversion of approximately 70%, with more than 60% of epi-units achieving ≥70% immunity, demonstrates that the national vaccination program has substantially contributed to PPR control by building population-level protective immunity with >60% vaccine effectiveness. Marked regional variation in seroconversion highlights the influence of ecological, managerial, and operational factors on vaccine performance, emphasizing the need for region-specific vaccination strategies, particularly in Northeastern and select Central and Western regions where immunity gaps persist. Despite these variations, the study confirms that the Sungri-96 vaccine is highly effective under field conditions when implemented through structured mass vaccination campaigns. Strengthening vaccination coverage, ensuring cold-chain integrity, and improving last-mile delivery systems will be essential to close residual immunity gaps and achieve uniform protection across all epi-units. Future seromonitoring and vaccine performance evaluations should be conducted at defined post-vaccination intervals to track population immunity dynamics and guide adaptive control strategies. Overall, the study confirms that the PPR Sungri-96 vaccine is an effective tool for achieving protective immunity under field conditions and provides strong scientific evidence supporting India’s progress toward PPR elimination under the NSP and alignment with the WOAH/FAO PPR-GEP 2030.

## Figures and Tables

**Table 1:** Evaluation of PPR Sungri-96 vaccine-induced seroconversion or PPRV antibody immune response in small ruminants (6–12 months) at the epidemiological unit level across different states of India.

Regional Zones and Name of the State/Union Territories	Make of PPR Vaccine Used in the Vaccination	Batch of PPR Vaccine Used in the Vaccination	Vaccination Coverage (%)	No. of Districts Covered	No. of Epi-units Included in the Survey	No. of Serum Samples Screened	No. of Samples Positive for PPRV Antibody	Overall Seroconversion/Vaccine Effectiveness (%)	Progress of Population/Herd Immunity (%)	Post-Vaccination % of Epi-Units Had >70% Immune Response	Pre-Vaccination * % of Epi-units Had >70% Seroprevalence	No. of Epi-Units Had >70% Seroprevalence/Sero surveillance in Pre-Vaccination	No. of Epi Units Included in the Survey in Seroprevalence/Sero surveillance in Pre-Vaccination
	Southern Zone												
Andhra Pradesh	Hester Biosciences Limited; IAHV&B	A49029; PPRV06/2223	94.9	26	120	1200	987	82.3 (95% CI: 80.0–84.3%)	78.1	77.5	37.3	38	102
Karnataka	IAH&VB;	7/C/2223; 2/V/2223	90.9	23	111	1102	809	73.4 (95% CI: 70.7–75.9%)	66.7	68.5	35.1	40	114
Keralam	Hester Biosciences Limited	NP	58.6	14	109	1010	625	61.9 (95% CI: 58.8–64.8%)	36.3	51.4	7.5	8	106
Puducherry	Hester Biosciences Limited	NP	~88	3	70	662	368	55.6 (95% CI: 51.8–59.3%)	48.9	41.4	8.1	6	74
Tamil Nadu	Hester Biosciences Limited	A49055, A49097	~90	31	120	1199	931	77.6 (95% CI: 75.2–79.9%)	69.8	72.5	25.9	30	116
Telangana	TSVBRI	Batch No. 11, 15, 7, 04/2324	91	33	120	1209	1108	91.6 (95% CI: 90.0–93.1%)	83.4	94.2	63.9	76	119
	Eastern Zone												
Bihar	Hester Biosciences Limited	A49007, A49069, A49075	99.6	38	115	1141	733	64.2 (95% CI: 61.4–67.0%)	63.9	59.1	22.4	19	95
Jharkhand	Hester Biosciences Limited	A49098, A49099, A50053, A49104	97.5	23	95	932	572	61.4 (95% CI: 58.2–64.4%)	59.9	54.7	31.8	30	88
Odisha	Hester Biosciences Limited	A49023, A49024, A49026, A49027	87.3	30	119	1125	865	76.9 (95% CI: 74.3–79.3%)	67.1	71.4	43.6	51	117
West Bengal	Hester Biosciences Limited	A49091, A49092, A49093, A49094	99.5	21	108	1078	859	79.7 (95% CI: 77.2–82.0%)	79.3	67.5	32.4	33	102
	Central Zone												
Chhattisgarh	Hester Biosciences Limited	A49068, A49067, A49069	90.4	27	118	1172	923	78.8 (95% CI: 76.3–81.0%)	71.2	75.4	19.5	23	118
Madhya Pradesh	Hester Biosciences Limited	A49081, A49084, A50092	96	35	108	1078	628	58.3 (95% CI: 55.3–61.2%)	56.0	51.9	18.8	19	101
	Western Zone												
Gujarat	Hester Biosciences Limited	A49039, A49040, A49049, A50042	99	32	120	1179	760	64.5 (95% CI: 61.7–67.1%)	63.9	50.8	19.6	22	112
Maharashtra	Hester Biosciences Limited	A49076, A49033, A49071, A49079, A49080	98.7	33	121	1109	834	75.2 (95% CI: 72.6–77.7%)	74.2	71.9	41.4	18	47
Rajasthan	Hester Biosciences Limited	NP	~85	27	94	930	602	64.7 (95% CI: 61.6–67.7%)	55.0	50	14.1	11	78
	Northern Zone												
Haryana	HVVI	A50058 Batch 1	~90	22	112	1116	813	72.8 (95% CI: 70.2–75.4%)	65.5	65.2	26.1	29	111
Himachal Pradesh	Hester Biosciences Limited	A49062, A49063	81	9	87	816	440	53.9 (95% CI: 50.5–57.3%)	43.7	37.9	15.9	14	88
Punjab	Hester Biosciences Limited	A49006, A49086	100	22	106	1049	711	67.8 (95% CI: 64.9–70.5%)	67.8	59.4	23.1	25	108
Uttar Pradesh	Hester Biosciences Limited	A49137	~84	56	125	1236	783	63.3 (95% CI: 60.6–66.0%)	53.2	58.3	28.3	8	22
	Northeastern Zone												
Assam	Hester Biosciences Limited	A49094, A49095	93	30	116	1145	798	69.7 (95% CI: 67.0–72.3%)	64.8	68.1	4.1	4	119
Manipur	Hester Biosciences Limited	NP	58.9	9	97	865	500	57.8 (95% CI: 54.5–61.1%)	34.0	28.9	0	0	96
Meghalaya	Hester Biosciences Limited	NP	~85	12	79	723	384	53.1 (95% CI: 49.5–56.7%)	45.1	44.3	5	5	100
Mizoram	Hester Biosciences Limited	A49094, A49733, 03PPR00123	75	5	72	625	464	74.2 (95% CI: 70.7–77.5%)	55.7	65.3	1.8	1	55
Sikkim	Hester Biosciences Limited	A49094	64.2	4	86	841	502	59.7 (95% CI: 56.3–63.0%)	38.3	52.3	0	0	99
Grand Total			87.4	565	2528	24,542	16,999	69.3 (95% CI: 68.7–69.8%)	60.6	61.4	21.9	510	2287

Note: Telangana State Veterinary Biological & Research Institute (TSVBRI), Hyderabad, Telangana, and the Institute of Animal Health & Veterinary Biologicals (IAH&VB), Karnataka, M/s Hester Biosciences Limited, and Haryana Veterinary Vaccine Institute (HVVI). NP—Details of the vaccine batch used were not provided. * Laboratory data generated under the separate baseline serosurvey in PPR-EP were used for comparison. The achievement of ≥70% protection levels in more than 50% of the surveyed epidemiological units (epi-units) indicates successful vaccine-induced seroconversion and field effectiveness, as per GCES guidelines for the PPR-GEP.

**Table 2:** Chi-square analysis of PPRV antibody immune response (seroconversion) in small ruminants across Indian states included in the PPR sungri-96 vaccine effectiveness study.

Category	Variables	No. of Serum Samples Screened	No. of Samples Positive	Seroconversion or Immune Response (%)	*χ* ^2^	*p*-Value
Across states	Different states	24,542	16,999	69.3 (95% CI: 68.7–69.8%)	1093.5	<0.05 *
Species	Sheep	5683	4297	75.6 (95% CI: 74.5–76.7%)	139.9	<0.05 *
	Goat	18,859	12,702	67.4 (95% CI: 66.7–68.0%)		
Sex	Male	6956	4645	66.7 (95% CI: 65.7–67.9%)	28.2	<0.05 *
	Female	17,586	12,354	70.2 (95% CI: 69.6–70.9%)		
Breed	Non-descriptive	23,654	16,369	69.2 (95% CI: 68.6–69.8%)	1.22	>0.05
	Crossbreed	888	630	70.0 (95% CI: 67.9–73.8%)		
Animal husbandry practice	Nomadic	2199	1531	69.6 (95% CI: 67.7–71.5%)	45.8	<0.05 *
	Intensive	692	500	72.2 (95% CI: 68.8–75.5%)		
	Semi-intensive	14,885	10,085	67.7 (95% CI: 67.0–68.5%)		
	Extensive	6766	4883	72.1 (95% CI: 71.1–73.2%)		

* significance at 95% confidence level.

**Table 3:** Post-vaccination seromonitoring of PPRV antibodies in small ruminants across India: distribution of epidemiological units by vaccine immune response levels (≤30%, 31–70%, and >70%).

Regional Zones/State	<30% Seroprevalence	31–70% Seroprevalence	>70% Seroprevalence	<30% Seroprevalence %	31–70% Seroprevalence %	>70% Seroprevalence %
	Epi-Unit Distribution Number			Epi-Unit Distribution Percentage		
Southern Zone						
Andhra Pradesh	3	24	93	2.5	20	77.5
Karnataka	10	25	76	9	22.5	68.5
Keralam	18	35	56	16.5	32.1	51.4
Puducherry	17	24	29	24.3	34.3	41.4
Tamil Nadu	1	32	87	0.8	26.7	72.5
Telangana	0	7	113	0	5.8	94.2
Eastern Zone						
Bihar	26	21	68	22.6	18.3	59.1
Jharkhand	23	20	52	24.2	21.1	54.7
Odisha	10	24	85	8.4	20.2	71.4
West Bengal	8	19	81	6.7	15.8	67.5
Central Zone						
Chhattisgarh	6	23	89	5.1	19.5	75.4
Madhya Pradesh	32	20	56	29.6	18.5	51.9
Western Zone						
Gujarat	14	45	61	11.7	37.5	50.8
Maharashtra	17	17	87	14	14	71.9
Rajasthan	14	33	47	14.9	35.1	50
Northern Zone						
Haryana	7	32	73	6.3	28.6	65.2
Himachal Pradesh	22	32	33	25.3	36.8	37.9
Punjab	18	25	63	17	23.6	59.4
Uttar Pradesh	27	28	70	22.5	23.3	58.3
Northeastern Zone						
Assam	19	18	79	16.4	15.5	68.1
Manipur	11	58	28	11.3	59.8	28.9
Meghalaya	31	13	35	39.2	16.5	44.3
Mizoram	4	21	47	5.6	29.2	65.3
Sikkim	23	18	45	26.7	20.9	52.3
Grand Total	361	614	1553	14.3	24.3	61.4

## Data Availability

The data supporting the findings of this study are available within the manuscript and the Supplementary Files. Additional datasets can be accessed upon reasonable request from the corresponding author at the ICAR-NIVEDI’s WOAH Reference Laboratory for PPR, Bengaluru.

## Supplementary Materials

The following supporting information can be downloaded at: https://www.mdpi.com/article/10.3390/v18101102/s1, Table S1: Details of the Indian States included in the PPR Sungri-96 Vaccine Effectiveness Study: Geographical Features and Small Ruminant Population. Table S2: Sampling plan for the comprehensive Post Vaccination Evaluation (PVE) strategic survey Protocol I approach as per WOAH/FAO’s GCES guidelines of the PPR-GEP. Table S3: Post-vaccination seromonitoring of PPRV antibodies in small ruminants across different states of India. Table S4: Details of the post-vaccination PPRV antibodies seroconversion in small ruminants in various districts of Andhra Pradesh. Table S5: Details of the post-vaccination PPRV antibodies seroconversion in small ruminants in various districts of Karnataka. Table S6: Details of the post-vaccination PPRV antibodies seroconversion in small ruminants in various districts of Keralam. Table S7: Details of the post-vaccination PPRV antibodies seroconversion in small ruminants in various districts of Puducherry. Table S8: Details of the post-vaccination PPRV antibodies seroconversion in small ruminants in various districts of Tamil Nadu. Table S9: Details of the post-vaccination PPRV antibodies seroconversion in small ruminants in various districts of Telangana. Table S10: Details of the post-vaccination PPRV antibodies seroconversion in small ruminants in various districts of Bihar. Table S11: Details of the post-vaccination PPRV antibodies seroconversion in small ruminants in various districts of Jharkhand. Table S12: Details of the post-vaccination PPRV antibodies seroconversion in small ruminants in various districts of Odisha. Table S13: Details of the post-vaccination PPRV antibodies seroconversion in small ruminants in various districts of West Bengal. Table S14: Details of the post-vaccination PPRV antibodies seroconversion in small ruminants in various districts of Chhattisgarh. Table S15: Details of the post-vaccination PPRV antibodies seroconversion in small ruminants in various districts of Madhya Pradesh. Table S16: Details of the post-vaccination PPRV antibodies seroconversion in small ruminants in various districts of Gujarat. Table S17: Details of the post-vaccination PPRV antibodies seroconversion in small ruminants in various districts of Maharashtra. Table S18: Details of the post-vaccination PPRV antibodies seroconversion in small ruminants in various districts of Rajasthan. Table S19: Details of the post-vaccination PPRV antibodies seroconversion in small ruminants in various districts of Haryana. Table S20: Details of the post-vaccination PPRV antibodies seroconversion in small ruminants in various districts of Himachal Pradesh. Table S21: Details of the post-vaccination PPRV antibodies Seroconversion in small ruminants in various districts of Punjab. Table S22: Details of the post-vaccination PPRV antibodies seroconversion in small ruminants in various districts of Uttar Pradesh. Table S23: Details of the post-vaccination PPRV antibodies seroconversion in small ruminants in various districts of Assam. Table S24: Details of the post-vaccination PPRV antibodies seroconversion in small ruminants in various districts of Manipur. Table S25: Details of the post-vaccination PPRV antibodies seroconversion in small ruminants in various districts of Meghalaya. Table S26: Details of the post-vaccination PPRV antibodies seroconversion in small ruminants in various districts of Mizoram. Table S27: Details of the post-vaccination PPRV antibodies seroconversion in small ruminants in various districts of Sikkim. Figure S1: Locations of surveyed epidemiological units-epi-units (villages) across 24 Indian states and union territories are depicted as dot in GIS-based maps of India. Figure S2: Distribution of PPR virus immune response antibodies in small ruminants across studies states, covering multiple epi-units after the first annual mass vaccination. Figure S3: Distribution of epi-units based on post-vaccination PPRV antibody immune response across studied states in India. These seromonitoring results for PPRV antibodies in small ruminants across surveyed states show the immunity status at the state level, highlighting the percentage prevalence of antibodies in small ruminants aged 6–12 months after first annual mass vaccination.
