# Supplementary material for: Effectiveness of the PPR Sungri-96 Vaccine in Sheep and Goats in India: A Nationwide Post-Vaccination Seromonitoring Study Towards PPR Elimination
Source: Viruses. 2026 Oct 6;18(10):1102. doi: 10.3390/v18101102 (PMC13709662; doi:10.3390/v18101102)
Supplement: Supplementary file 1 [file viruses-18-01102-s001.zip › viruses-4512738-supplementary.pdf]

Supplementary Documents

Title: Effectiveness of the PPR Sungri-96 Vaccine in Sheep and Goats in India: A Nationwide Post-Vaccination Seromonitoring Study Towards PPR Elimination

Table S1: Details of the Indian States included in the PPR Sungri 96 Vaccine Effectiveness Study: Geographical Features and Small Ruminant Population

Region	State	Geographical Features	Transboundary nature of Risk with Neighbouring Countries)	Area (Sq. km)	Sheep* Population (million)	Goat* Population (million)	Total* Population (million)
Southern	Andhra Pradesh	Southeastern coast of India	None	1,60,205	17.63	5.52	23.15
	Telangana	Deccan Plateau	None	1,12,077	19.06	4.93	23.99
	Karnataka	Southern peninsular state with diverse terrain	None	1,91,791	11.05	6.17	17.22
	Tamil Nadu	Plains with significant river systems	None	1,30,058	4.5	9.89	14.39
	Puducherry	Coastal plains	None	492	0.002	0.073	0.075
	Keralam	Coastal and hilly terrain	None	38863	0.1	1.47	1.57
Eastern	Odisha	Plains and forested areas	Bangladesh	1,55,707	1.28	6.39	7.67
	Bihar	Fertile plains with river systems	Nepal, Bangladesh	94,163	0.21	12.82	13.03
	Jharkhand	Plateau and forested terrain	None	79716	0.15	5.09	5.24
	West Bengal	River delta plains	Bangladesh	88752	0.04	11.09	11.13
Central	Chhattisgarh	Predominantly forested	None	1,35,191	0.18	4.01	4.19
	Madhya Pradesh	Large geographical state with plateaus	None	3,08,350	0.32	11.06	11.38
Western	Gujarat	Plains and semi-arid regions	Pakistan	1,96,024	1.79	4.87	6.66
	Maharashtra	Plateau and coastal diversity	None	307713	2.68	10.6	13.28
	Rajasthan	Arid and semi-arid desert	Pakistan	342239	9.12	10.02	19.14
Northern	Punjab	Fertile plains	Pakistan	50,362	0.09	0.35	0.44
	Haryana	Fertile plains with semi-arid regions	Pakistan	44,212	0.29	0.33	0.62
	Uttar Pradesh	Fertile plains along Ganges	Nepal	243286	0.36	13.91	14.27
	Himachal Pradesh	Mountainous	China	55673	0.8	0.68	1.48
Northeastern	Mizoram	Rugged, hilly terrain	Bangladesh, Myanmar	21,081	0.01	0.13	0.14
	Assam	River valleys and hills	Bhutan, Bangladesh	78438	0.01	4.57	4.58
	Manipur	Hilly and forested	Myanmar	22327	0.03	0.28	0.31
	Meghalaya	Hilly plateaus	Bangladesh	22429	0.01	0.49	0.5
	Sikkim	Mountainous	Nepal, China	7096	0.002	0.04	0.042
**Total**				28,86,245	69.7	124.8	194.5

1. *** Population as per the 20th Livestock Census, 2019, DAHD, GoI [21].** 21. Anonymous. India, Ministry of Fisheries, Animal Husbandry and Dairying, Department of Animal Husbandry and Dairying. 20th Livestock Census-2019: All India Report. Government of India, 2019, dahd.gov.in/schemes/programmes/animal-husbandry-statistics

Table S2: Sampling plan for the comprehensive Post Vaccination Evaluation (PVE) strategic survey Protocol I approach as per WOAH/FAO's GCES guidelines of the PPR-GEP

Country	India	Remarks and Samples size estimation
State	Each administrative of the federal state of India. For eg. Andhra Pradesh	The study area is purposefully selected as the state, as animal husbandry subject is federal state specific in India
Villages /Epiunits	Random selection of 108-120 villages in the study area/region- for each state	Villages as an epidemiological unit (epi-unit) are to be selected randomly concerning the size of the population.<br>Before the stratified randomization of villages, the trimming of the sampling frame (source of villages from which samples are to be drawn) was employed for 500 animals or 100 as per exiting population in particular state per village as an inclusion criterion to have a substantial population
Household or Flocks	3 or 4 flocks in the village by random/ convenient selection	Based on the small ruminant population, a maximum of 3-4 animals from each flock for stratum (age group between 6-12 months) were selected conveniently in each epidemiological unit or village
Animal Units for Post-vaccination -Vaccine Effectiveness Survey	Post-vaccination immune response -Seroconversion	Total No. of samples to be tested after implementation of first round of mass vaccination in each state of India under PPR-EP. Minimum animal samples size = 108 x 9 = 972 samples or Maximum animal samples size = 108 x 10= 1080 samples /geographical area.<br>Post-vaccination vaccine Effectiveness (seroconversion) was assessed using 10 serum samples collected from small ruminants aged 6–12 months within each epidemiological unit.

Table S3. Post-vaccination seromonitorng of PPR in small ruminants across different states of India during 2023−2024

State/UTs	No. of Districts in the State	No. of Taluks in the State	No. of Village/ Epi-Units	No. of serum samples screened	No. of samples positive in ELISA	Seroconversion (%)	No. of serum samples screened	No. of samples positive in ELISA	Seroconversion (%)	No. of serum samples screened	No. of samples positive in ELISA	Seroconversion (%)
				Sheep			Goat			Total		
**Southern Zone**												
**Andhra Pradesh**	26	115	120	1018	847	83.2	182	140	76.9	1200	987	82.3
**Karnataka**	23	65	111	671	483	72	431	326	75.6	1102	809	73.4
**Keralam**	14	51	109	-	-	-	1010	625	61.9	1010	625	61.9
**Puducherry**	3	9	70	34	16	47.1	628	352	56.1	662	368	55.6
**Tamil Nadu**	31	94	120	290	205	70.7	909	726	79.9	1199	931	77.6
**Telangana**	33	103	120	1093	999	91.4	116	109	94	1209	1108	91.6
**Eastern Zone**												
**Bihar**	38	103	115	87	55	63.2	1054	678	64.3	1141	733	64.2
**Jharkhand**	23	74	95	31	24	77.4	901	548	60.8	932	572	61.4
**Odisha**	30	115	119	178	127	71.3	947	738	77.9	1125	865	76.9
**West Bengal**	21	86	108	40	28	70	1038	831	80.1	1078	859	79.7
**Central Zone**												
**Chhattisgarh**	27	78	118	452	384	85	720	539	74.9	1172	923	78.8
**Madhya Pradesh**	35	87	108	54	36	66.7	1024	592	57.8	1078	628	58.3
**Western Zone**												
**Gujarat**	32	85	120	274	180	65.7	905	580	64.1	1179	760	64.5
**Maharashtra**	33	99	121	130	108	83.1	979	726	74.2	1109	834	75.2
**Rajasthan**	27	81	94	10	6	60	920	596	64.8	930	602	64.7
**Northern Zone**												
**Haryana**	22	54	112	681	466	68.4	435	347	79.8	1116	813	72.8
**Himachal Pradesh**	9	44	87	335	162	48.4	481	278	57.8	816	440	53.9
**Punjab**	22	59	106	153	97	63.4	896	614	68.5	1049	711	67.8
**Uttar Pradesh**	56	101	125	64	39	60.9	1172	744	63.5	1236	783	63.3
**Northeastern Zone**												
**Assam**	30	79	116	76	29	38.2	1069	769	71.9	1145	798	69.7
**Manipur**	9	37	97	5	1	20	860	499	58	865	500	57.8
**Meghalaya**	12	26	79	-	-	-	723	384	53.1	723	384	53.1
**Mizoram**	5	15	72	3	3	100	622	461	74.1	625	464	74.2
**Sikkim**	4	15	86	4	2	50	837	500	59.7	841	502	59.7
**Grand Total**	**565**	**1675**	**2528**	**5683**	**4297**	**75.6**	**18859**	**12702**	**67.4**	**24542**	**16999**	**69.3**
***Chi-squared value ($\chi2$): p-value and Seroconversion at 95% CI**				**$\chi2$: 487.9, P <0.005**		**CI 95%: (74 to 77)**	**$\chi2$: 613.2, P <0.005**		**CI 95%: (67 to 68)**	**$\chi2$: 1093.5, P <0.005**		**CI 95%: (69 to 70)**

*$\chi2$ value was not calculated for Manipur, Meghalaya, Sikkim, Mizoram and Keralam while analysis association across sheep populations as their values were less than 5

CI—confidence interval

Table S4: Details of the post-vaccination PPRV antibodies seroconversion in small ruminants in various districts of Andhra Pradesh

Name of District	No. of Taluks in the district	No. of Village/ Epi-Units	No. of serum samples screened	No. of samples positive in ELISA	Seroconversion (%)	No. of serum samples screened	No. of samples positive in ELISA	Seroconversion (%)	No. of serum samples screened	No. of samples positive in ELISA	Seroconversion (%)
			Sheep			Goat			Total		
Chittoor	4	4	40	37	92.5	-	-	-	40	37	92.5
East Godavari	3	4	40	40	100	-	-	-	40	40	100
Guntur	3	3	30	23	76.7	-	-	-	30	23	76.7
Krishna	4	4	40	40	100	-	-	-	40	40	100
Kurnool	4	4	36	36	100	4	4	100	40	40	100
Prakasam	10	10	100	88	88	-	-	-	100	88	88
Srikakulam	10	11	71	51	71.8	39	27	69.2	110	78	70.9
Vizianagaram	7	7	58	48	82.8	12	11	91.7	70	59	84.3
West Godavari	5	5	34	27	79.4	16	7	43.8	50	34	68
Y.S.R.	4	4	40	35	87.5	-	-	-	40	35	87.5
Eluru	5	6	33	26	78.8	27	24	88.9	60	50	83.3
Ananthapuramu	4	4	40	28	70	-	-	-	40	28	70
Annamayya	4	4	40	36	90	-	-	-	40	36	90
Kakinada	4	4	40	34	85	-	-	-	40	34	85
Nandyal	3	3	30	30	100	-	-	-	30	30	100
Palnadu	3	4	40	27	67.5	-	-	-	40	27	67.5
Sri Potti Sriramulu Nellore	6	6	60	43	71.7	-	-	-	60	43	71.7
Sri Sathya Sai	4	4	40	37	92.5	-	-	-	40	37	92.5
Tirupati	4	4	40	33	82.5	-	-	-	40	33	82.5
Anakapalli	4	4	19	19	100	21	21	100	40	40	100
Alluri Sitharama Raju	4	4	14	4	28.6	26	15	57.7	40	19	47.5
Bapatla	2	2	20	8	40	-	-	-	20	8	40
Dr. B.R. Ambedkar Konaseema	4	4	40	33	82.5	-	-	-	40	33	82.5
NTR	4	4	35	34	97.1	5	5	100	40	39	97.5
Parvathipuram Manyam	3	3	24	16	66.7	6	3	50	30	19	63.3
Visakhapatnam	3	4	14	14	100	26	23	88.5	40	37	92.5
**Grand Total**	**115**	**120**	**1018**	**847**	**83.2**	**182**	**140**	**76.9**	**1200**	**987**	**82.3**
**Seroconversion at 95% CI**					**CI 95%: (81 to 85)**			**CI 95%: (71 to 83)**			**CI 95%: (80 to 84)**

Table S5: Details of the post-vaccination PPRV antibodies seroconversion in small ruminants in various districts of Karnataka

Name of District	No. of Taluks in the District	No. of Village/ Epi-Units	No. of serum samples screened	No. of samples positive in ELISA	Seroconversion (%)	No. of serum samples screened	No. of samples positive in ELISA	Seroconversion (%)	No. of serum samples screened	No. of samples positive in ELISA	Seroconversion (%)
			Sheep			Goat			Total		
Bagalkot	2	4	31	30	96.8	6	6	100	37	36	97.3
Bengaluru Urban	3	5	28	24	85.7	22	18	81.8	50	42	84
Bijapur	2	3	13	10	76.9	17	16	94.1	30	26	86.7
Chamarajanagar	1	5	33	30	90.9	17	13	76.5	50	43	86
Chikballapur	4	8	67	51	76.1	12	10	83.3	79	61	77.2
Chikmagalur	3	6	45	24	53.3	14	7	50	59	31	52.5
Davangere	2	2	18	12	66.7	2	2	100	20	14	70
Dharwad	1	2	11	11	100	9	9	100	20	20	100
Gadag	2	2	15	12	80	5	4	80	20	16	80
Gulbarga	6	6	7	6	85.7	51	50	98	58	56	96.6
Hassan	5	12	73	42	57.5	47	27	57.4	120	69	57.5
Haveri	3	5	34	30	88.2	16	16	100	50	46	92
Kolar	5	8	63	38	60.3	16	9	56.3	79	47	59.5
Koppal	4	5	45	41	91.1	5	5	100	50	46	92
Mandya	3	4	19	9	47.4	21	7	33.3	40	16	40
Mysore	4	7	30	23	76.7	40	35	87.5	70	58	82.9
Ramanagara	3	5	30	17	56.7	20	12	60	50	29	58
Shimoga	3	8	46	20	43.5	34	22	64.7	80	42	52.5
Uttar Kannada	2	5	11	9	81.8	39	33	84.6	50	42	84
Yadgir	2	2	16	14	87.5	4	4	100	20	18	90
Dakshina Kannada	1	2	-	-	-	20	10	50	20	10	50
Bider	1	2	6	2	33.3	14	11	78.6	20	13	65
Raichur	3	3	30	28	93.3	-	-	-	30	28	93.3
**Grand Total**	**64**	**111**	**671**	**483**	**72**	**431**	**326**	**75.6**	**1102**	**809**	**73.4**
**Seroconversion at 95% CI**					**CI 95%: (69 to 75)**			**CI 95%: (72 to 80)**			**CI 95%: (71 to 76)**

Table S6: Details of the post-vaccination PPRV antibodies seroconversion in small ruminants in various districts of Keralam

Name of District	No. of Taluks in the State	No. of Village/ Epi-Units	Sheep: No. of serum samples screened	Sheep: No. of samples positive in ELISA	Sheep: Seroconversion (%)	Goat: No. of serum samples screened	Goat: No. of samples positive in ELISA	Goat: Seroconversion (%)	Total: No. of serum samples screened	Total: No. of samples positive in ELISA	Total: Seroconversion (%)
Alappuzha	3	7	-	-	-	64	42	65.6	64	42	65.6
Ernakulam	6	10	-	-	-	93	68	73.1	93	68	73.1
Idukki	3	5	-	-	-	44			44	0	0
Kannur	4	9	-	-	-	81	66	81.5	81	66	81.5
Kasaragod	4	5	-	-	-	46	35	76.1	46	35	76.1
Kollam	3	8	-	-	-	75	10	13.3	75	10	13.3
Kottayam	3	8	-	-	-	73	59	80.8	73	59	80.8
Kozhikode	2	9	-	-	-	83	72	86.7	83	72	86.7
Malappuram	7	13	-	-	-	119	88	73.9	119	88	73.9
Palakkad	4	9	-	-	-	88	22	25	88	22	25
Pathanamthitta	3	5	-	-	-	45	36	80	45	36	80
Thiruvananthapuram	4	9	-	-	-	85	39	45.9	85	39	45.9
Thrissur	4	9	-	-	-	84	64	76.2	84	64	76.2
Wayanad	1	3	-	-	-	30	24	80	30	24	80
**Grand Total**	**51**	**109**	-	-	-	**1010**	**625**	**61.9**	**1010**	**625**	**61.9**
**Seroconversion at 95% CI**					-			**CI 95%: (59 to 65)**			**CI 95%: (59 to 65)**

Table S7: Details of the post-vaccination PPRV antibodies seroconversion in small ruminants in various districts of Puducherry

Name of District	No. of Taluks in the State	No. of Village/ Epi-Units	No. of serum samples screened	No. of samples positive in ELISA	Seroconversion (%)	No. of serum samples screened	No. of samples positive in ELISA	Seroconversion (%)	No. of serum samples screened	No. of samples positive in ELISA	Seroconversion (%)
			Sheep			Goat			Total		
Karaikal	3	21	4	2	50	206	184	89.3	210	186	88.6
Mahe	1	4	-	-	-	33	13	39.4	33	13	39.4
Pondicherry	5	45	30	14	46.7	389	155	39.8	419	169	40.3
**Grand Total**	**9**	**70**	**34**	**16**	**47.1**	**628**	**352**	**56.1**	**662**	**368**	**55.6**
**Seroconversion at 95% CI**					**CI 95%: (30 to 64)**			**CI 95%: (52 to 60)**			**CI 95%: (52 to 59)**

Table S8: Details of the post-vaccination PPRV antibodies seroconversion in small ruminants in various districts of Tamil Nadu

Name of District	No. of Taluks in the District	No. of Village/ Epi-Units	No. of serum samples screened	No. of samples positive in ELISA	Seroconversion (%)	No. of serum samples screened	No. of samples positive in ELISA	Seroconversion (%)	No. of serum samples screened	No. of samples positive in ELISA	Seroconversion (%)
			Sheep			Goat			Total		
Ariyalur	1	2	3	3	100	17	14	82.4	20	17	85
Coimbatore	2	4	8	4	50	32	25	78.1	40	29	72.5
Cuddalore	4	5	3	3	100	47	38	80.9	50	41	82
Dharmapuri	3	3	6	5	83.3	24	16	66.7	30	21	70
Dindigul	3	4	15	10	66.7	25	18	72	40	28	70
Erode	3	5	17	11	64.7	33	28	84.8	50	39	78
Kanchipuram	5	5	21	16	76.2	29	19	65.5	50	35	70
Kanniyakumari	2	4	-	-	-	40	15	37.5	40	15	37.5
Karur	3	3	-	-	-	30	29	96.7	30	29	96.7
Krishnagiri	3	3	16	11	68.8	14	13	92.9	30	24	80
Madurai	4	4	15	4	26.7	25	23	92	40	27	67.5
Nagapattinam	3	3	-	-	-	30	26	86.7	30	26	86.7
Namakkal	3	4	10	5	50	30	21	70	40	26	65
Perambalur	1	1	10	9	90	-	-	-	10	9	90
Pudukkottai	4	4	10	7	70	30	23	76.7	40	30	75
Ramanathapuram	2	3	-	-	-	30	18	60	30	18	60
Salem	4	5	8	6	75	42	30	71.4	50	36	72
Sivaganga	3	4	8	8	100	32	32	100	40	40	100
Thanjavur	4	5	-	-	-	50	46	92	50	46	92
The Nilgiris	3	3	10	5	50	20	13	65	30	18	60
Theni	2	3	7	5	71.4	23	21	91.3	30	26	86.7
Thiruvallur	3	4	8	2	25	32	21	65.6	40	23	57.5
Thiruvarur	1	3	-	-	-	30	29	96.7	30	29	96.7
Tiruchirappalli	3	4	4	3	75	36	34	94.4	40	37	92.5
Tirunelveli	3	5	22	19	86.4	28	19	67.9	50	38	76
Tiruppur	3	3	12	10	83.3	18	16	88.9	30	26	86.7
Tiruvannamalai	3	5	12	8	66.7	38	31	81.6	50	39	78
Tuticorin	3	4	21	12	57.1	19	13	68.4	40	25	62.5
Vellore	5	5	20	20	100	30	29	96.7	50	49	98
Villupuram	5	7	10	8	80	60	52	86.7	70	60	85.7
Virudhunagar	3	3	14	11	78.6	15	14	93.3	29	25	86.2

**Grand Total**	**94**	**120**	**290**	**205**	**70.7**	**909**	**726**	**79.9**	**1199**	**931**	**77.6**
**Seroconversion at 95% CI**					**CI 95%: (65 to 76)**			**CI 95%: (77 to 82)**			**CI 95%: (75 to 80)**

Table S9: Details of the post-vaccination PPRV antibodies seroconversion in small ruminants in various districts of Telangana

Name of District	No. of Taluks in the District	No. of Village/ Epi-Units	No. of serum samples screened	No. of samples positive in ELISA	Seroconversion (%)	No. of serum samples screened	No. of samples positive in ELISA	Seroconversion (%)	No. of serum samples screened	No. of samples positive in ELISA	Seroconversion (%)
			Sheep			Goat			Total		
Adilabad	3	4	18	17	94.4	22	21	95.5	40	38	95
Hyderabad	1	1	2	1	50	8	6	75	10	7	70
Jogulamba Gadwal	2	3	26	26	100	4	4	100	30	30	100
Kamareddy	5	5	44	42	95.5	6	6	100	50	48	96
Karimnagar	3	3	28	25	89.3	2	1	50	30	26	86.7
Khammam	5	5	42	42	100	8	8	100	50	50	100
Mahabubabad	3	3	23	22	95.7	7	6	85.7	30	28	93.3
Mancherial	3	4	35	35	100	5	5	100	40	40	100
Medak	4	4	39	36	92.3	-	-	-	39	36	92.3
Medchal Malkajgiri	2	2	20	14	70	-	-	-	20	14	70
Nagarkurnool	3	4	40	39	97.5	-	-	-	40	39	97.5
Nalgonda	5	6	60	57	95	-	-	-	60	57	95
Nirmal	4	4	40	40	100	-	-	-	40	40	100
Peddapalli	3	3	30	27	90	-	-	-	30	27	90
Sangareddy	7	7	70	54	77.1	-	-	-	70	54	77.1
Siddipet	4	4	40	23	57.5	-	-	-	40	23	57.5
Suryapet	3	4	40	38	95	-	-	-	40	38	95
Vikarabad	3	5	50	46	92	-	-	-	50	46	92
Wanaparthy	2	3	30	29	96.7	-	-	-	30	29	96.7
Warangal Rural	1	3	30	29	96.7	-	-	-	30	29	96.7
Warangal Urban	2	3	30	29	96.7	-	-	-	30	29	96.7
Yadadri Bhuvanagiri	4	4	40	29	72.5	-	-	-	40	29	72.5
Bhadradri	4	4	27	27	100	13	11	84.6	40	38	95
Jagtial	3	3	27	24	88.9	3	3	100	30	27	90
Janagoan	2	3	25	24	96	5	5	100	30	29	96.7
Jayashankar Bhupalpally	4	4	39	39	100	1	1	100	40	40	100
KB Asifabad	2	4	14	14	100	26	26	100	40	40	100
Mahabubnagar	3	3	38	38	100	2	2	100	40	40	100
Narayanpet	1	1	9	8	88.9	1	1	100	10	9	90
Nizamabed	4	5	50	50	100	-	-	-	50	50	100

R.Sircilla	2	3	30	25	83.3	-	-	-	30	25	83.3
Rangareddy	5	5	50	43	86	-	-	-	50	43	86
Mugulu	1	1	7	7	100	3	3	100	10	10	100
**Grand Total**	**103**	**120**	**1093**	**999**	**91.4**	**116**	**109**	**94**	**1209**	**1108**	**91.6**
**Seroconversion at 95% CI**					**CI 95%: (90 to 93)**			**CI 95%: (90 to 98)**			**CI 95%: (90 to 93)**

Table S10: Details of the post-vaccination PPRV antibodies seroconversion in small ruminants in various districts of Bihar

Name of District	No. of Taluks in the District	No. of Village/ Epi-Units	No. of serum samples screened	No. of samples positive in ELISA	Seroconversion (%)	No. of serum samples screened	No. of samples positive in ELISA	Seroconversion (%)	No. of serum samples screened	No. of samples positive in ELISA	Seroconversion (%)
			Sheep			Goat			Total		
Arwal	1	1	5	5	100	5	0	0	10	5	50
Aurangabad	4	4	5	5	100	35	25	71.4	40	30	75
Banka	3	5	-	-	-	47	26	55.3	47	26	55.3
Bhojpur	3	3	11	11	100	19	9	47.4	30	20	66.7
Buxar	2	3	13	13	100	16	3	18.8	29	16	55.2
Darbhanga	2	2	-	-	-	20	3	15	20	3	15
Jamui	4	4	-	-	-	40	36	90	40	36	90
Jehanabad	2	3	-	-	-	30	30	100	30	30	100
Kaimur (Bhabua)	3	3	24	7	29.2	6	5	83.3	30	12	40
Katihar	4	4	-	-	-	40	36	90	40	36	90
Lakhisarai	2	2	-	-	-	20	19	95	20	19	95
Madhubani	3	3	-	-	-	30	0	0	30	0	0
Nalanda	2	3	2	0	0	27	1	3.7	29	1	3.4
Patna	3	3	-	-	-	30	30	100	30	30	100
Samastipur	3	3	-	-	-	30	24	80	30	24	80
Sitamarhi	3	3	-	-	-	30	30	100	30	30	100
Siwan	4	4	-	-	-	40	29	72.5	40	29	72.5
Araria	2	2	-	-	-	20	14	70	20	14	70
Bhagalpur	3	3	-	-	-	29	28	96.6	29	28	96.6
Gaya	5	5	8	4	50	42	24	57.1	50	28	56
Gopalganj	4	4	-	-	-	40	16	40	40	16	40
Kishanganj	2	3	-	-	-	30	28	93.3	30	28	93.3
Madhepura	2	2	-	-	-	20	0	0	20	0	0
Munger	3	3	-	-	-	30	30	100	30	30	100
Muzaffarpur	3	3	-	-	-	29	0	0	29	0	0
Nawada	3	3	-	-	-	30	30	100	30	30	100
Purbi Champaran	3	4	-	-	-	39	31	79.5	39	31	79.5
Purnia	3	4	-	-	-	40	34	85	40	34	85
Rohtas	4	4	17	8	47.1	23	14	60.9	40	22	55
Saran	2	3	2	2	100	28	5	17.9	30	7	23.3
Sheikhpura	1	1	-	-	-	10	10	100	10	10	100

Supaul	2	3	-	-	-	29	20	69	29	20	69
Vaishali	3	4	-	-	-	40	6	15	40	6	15
Begusarai	3	3	-	-	-	30	18	60	30	18	60
Khagaria	1	1	-	-	-	10	4	40	10	4	40
Pashchim Champaran	3	3	-	-	-	30	27	90	30	27	90
Saharsa	11	3	-	-	-	30	25	83.3	30	25	83.3
Sheohar	5	1	-	-	-	10	8	80	10	8	80
**Grand Total**	**87**	**115**	**87**	**55**	**63.2**	**1054**	**678**	**64.3**	**1141**	**733**	**64.2**
**Seroconversion at 95% CI**					**CI 95%: (53 to 73)**			**CI 95%: (61 to 67)**			**CI 95%: (61 to 67)**

Table S11: Details of the post-vaccination PPRV antibodies seroconversion in small ruminants in various districts of Jharkhand

Name of District	No. of Taluks in the State	No. of Village/ Epi-Units	Sheep: No. of serum samples screened	Sheep: No. of samples positive in ELISA	Sheep: Seroconversion (%)	Goat: No. of serum samples screened	Goat: No. of samples positive in ELISA	Goat: Seroconversion (%)	Total: No. of serum samples screened	Total: No. of samples positive in ELISA	Total: Seroconversion (%)
Deoghar	3	5	-	-	-	57	27	47.4	57	27	47.4
Dhanbad	4	5	-	-	-	49	49	100	49	49	100
East Singhbum	4	7	1	1	100	69	36	52.2	70	37	52.9
Godda	3	5	-	-	-	49	28	57.1	49	28	57.1
Gumla	3	4	1	1	100	38	36	94.7	39	37	94.9
Khunti	2	3	8	5	62.5	22	22	100	30	27	90
Palamu	6	6	1	0	0	53	10	18.9	54	10	18.5
Ramgarh	2	3	-	-	-	29	28	96.6	29	28	96.6
Ranchi	5	5	3	3	100	47	29	61.7	50	32	64
West Singhbhum	3	5	6	5	83.3	44	19	43.2	50	24	48
Garhwa	3	4	-	-	-	40	14	35	40	14	35
Hazaribagh	4	5	-	-	-	45	44	97.8	45	44	97.8
Latehar	3	3	-	-	-	30	16	53.3	30	16	53.3
Chatra	5	5	-	-	-	50	34	68	50	34	68
Koderma	2	2	-	-	-	20	9	45	20	9	45
Lohardaga	3	3	-	-	-	29	18	62.1	29	18	62.1
Saraikela Kharsawan	2	2	3	3	100	17	13	76.5	20	16	80
Jamtara	3	4	-	-	-	40	22	55	40	22	55
Dumka	4	6	2	2	100	56	38	67.9	58	40	69
Bokaro	1	1	-	-	-	10	4	40	10	4	40
Simdega	2	3	-	-	-	28	17	60.7	28	17	60.7
Sahebganj	2	3	-	-	-	28	7	25	28	7	25
Giridih	5	6	6	4	66.7	51	28	54.9	57	32	56.1
**Grand Total**	**74**	**95**	**31**	**24**	**77.4**	**901**	**548**	**60.8**	**932**	**572**	**61.4**
**Seroconversion at 95% CI**					**CI 95%: (63 to 92)**			**CI 95%: (58 to 64)**			**CI 95%: (58 to 64)**

Table S12: Details of the post-vaccination PPRV antibodies seroconversion in small ruminants in various districts of Odisha

Name of District	No. of Taluks in the District	No. of Village/ Epi-Units	No. of serum samples screened	No. of samples positive in ELISA	Seroconversion (%)	No. of serum samples screened	No. of samples positive in ELISA	Seroconversion (%)	No. of serum samples screened	No. of samples positive in ELISA	Seroconversion (%)
			Sheep			Goat			Total		
Balangir	4	4	6	6	100	27	27	100	33	33	100
Baleshwar	6	7	-	-	-	68	37	54.4	68	37	54.4
Bargarh	2	3	-	-	-	29	21	72.4	29	21	72.4
Bhadrak	2	2	-	-	-	19	18	94.7	19	18	94.7
Boudh	2	3	7	6	85.7	20	20	100	27	26	96.3
Cuttack	5	5	9	2	22.2	37	10	27	46	12	26.1
Deogarh	3	3	-	-	-	26	0	0	26	0	0
Dhenkanal	3	3	6	6	100	24	21	87.5	30	27	90
Gajapati	4	4	-	-	-	36	30	83.3	36	30	83.3
Ganjam	6	7	34	20	58.8	36	23	63.9	70	43	61.4
Jagatsinghapur	2	2	-	-	-	18	17	94.4	18	17	94.4
Jajapur	3	3	-	-	-	29	29	100	29	29	100
Jharsuguda	1	1	-	-	-	10	8	80	10	8	80
Kalahandi	4	5	15	13	86.7	33	28	84.8	48	41	85.4
Kandhamal	4	5	-	-	-	49	49	100	49	49	100
Kendrapara	3	5	8	5	62.5	27	22	81.5	35	27	77.1
Kendujhar	3	4	-	-	-	38	27	71.1	38	27	71.1
Khordha	4	4	6	4	66.7	33	27	81.8	39	31	79.5
Koraput	4	5	29	24	82.8	21	19	90.5	50	43	86
Malkangiri	12	3	-	-	-	30	28	93.3	30	28	93.3
Mayurbhanj	9	9	4	1	25	81	64	79	85	65	76.5
Nabarangpur	3	3	15	12	80	13	11	84.6	28	23	82.1
Nayagarh	3	3	8	8	100	22	22	100	30	30	100
Nuapada	2	3	10	4	40	20	18	90	30	22	73.3
Puri	4	4	8	3	37.5	29	19	65.5	37	22	59.5
Rayagada	4	5	6	6	100	44	44	100	50	50	100
Sambalpur	2	3	-	-	-	30	24	80	30	24	80
Sonepur	3	3	7	7	100	21	19	90.5	28	26	92.9
Sundargarh	5	5	-	-	-	48	36	75	48	36	75
Angul	3	3	-	-	-	29	20	69	29	20	69
**Grand Total**	**115**	**119**	**178**	**127**	**71.3**	**947**	**738**	**77.9**	**1125**	**865**	**76.9**
**Seroconversion at 95% CI**					**CI 95%: (65 to 78)**			**CI 95%: (75 to 81)**			**CI 95%: (74 to 79)**

Table S13: Details of the post-vaccination PPRV antibodies seroconversion in small ruminants in various districts of West Bengal

Name of District	No. of Taluks in the State	No. of Village/ Epi-Units	No. of serum samples screened	No. of samples positive in ELISA	Seroconversion (%)	No. of serum samples screened	No. of samples positive in ELISA	Seroconversion (%)	No. of serum samples screened	No. of samples positive in ELISA	Seroconversion (%)
			Sheep			Goat			Total		
24 Paraganas North	3	4	-	-	-	37	26	70.3	37	26	70.3
24 Paraganas South	5	5	-	-	-	50	42	84	50	42	84
Alipurduar	1	1	-	-	-	10	10	100	10	10	100
Bankura	8	9	4	4	100	86	67	77.9	90	71	78.9
Bardhaman	5	7	4	4	100	66	59	89.4	70	63	90
Birbhum	7	7	7	6	85.7	63	45	71.4	70	51	72.9
Darjeeling	2	3	-	-	-	30	21	70	30	21	70
Dinajpur Dakshin	4	5	-	-	-	47	26	55.3	47	26	55.3
Dinajpur Uttar	3	5	-	-	-	49	40	81.6	49	40	81.6
Hooghly	4	4	-	-	-	38	23	60.5	38	23	60.5
Howrah	2	2	-	-	-	20	14	70	20	14	70
Jalpaiguri	2	3	-	-	-	29	27	93.1	29	27	93.1
Jhargram	5	7	7	4	57.1	63	61	96.8	70	65	92.9
Kalimpong	1	1	-	-	-	10	8	80	10	8	80
Maldah	5	5	-	-	-	50	50	100	50	50	100
Medinipur East	6	9	2	2	100	88	54	61.4	90	56	62.2
Medinipur West	8	14	-	-	-	138	121	87.7	138	121	87.7
Murshidabad	4	5	-	-	-	50	49	98	50	49	98
Nadia	3	3	-	-	-	30	22	73.3	30	22	73.3
Paschim Bardhaman	3	3	-	-	-	30	29	96.7	30	29	96.7
Purulia	6	7	16	8	50	54	37	68.5	70	45	64.3
**Grand Total**	**87**	**109**	**40**	**28**	**70**	**1038**	**831**	**80.1**	**1078**	**859**	**79.7**
**Seroconversion at 95% CI**					**CI 95%: (56 to 84)**			**CI 95%: (78 to 82)**			**CI 95%: (77 to 82)**

Table S14: Details of the post-vaccination PPRV antibodies seroconversion in small ruminants in various districts of Chhattisgarh

Name of District	No. of Taluks in the District	No. of Village/ Epi-Units	Sheep: No. of serum samples screened	Sheep: No. of samples positive in ELISA	Sheep: Seroconversion (%)	Goat: No. of serum samples screened	Goat: No. of samples positive in ELISA	Goat: Seroconversion (%)	Total: No. of serum samples screened	Total: No. of samples positive in ELISA	Total: Seroconversion (%)
Balod	2	3	16	16	100	14	14	100	30	30	100
Baloda Bazar	3	5	-	-	-	49	47	95.9	49	47	95.9
Balrampur	3	4	-	-	-	40	21	52.5	40	21	52.5
Bastar	4	4	9	9	100	31	31	100	40	40	100
Bemetara	2	3	-	-	-	30	12	40	30	12	40
Bilaspur	4	5	-	-	-	50	28	56	50	28	56
Dantewada	2	3	-	-	-	30	22	73.3	30	22	73.3
Dhamtari	3	5	-	-	-	50	21	42	50	21	42
Durg	2	4	6	6	100	34	30	88.2	40	36	90
Gariyaband	2	4	11	7	63.6	29	28	96.6	40	35	87.5
Janjgir-Champa	4	5	11	11	100	38	35	92.1	49	46	93.9
Jashpur	4	5	-	-	-	49	45	91.8	49	45	91.8
Kabirdham (Kawardha)	2	5	1	1	100	48	47	97.9	49	48	98
Kanker	4	5	-	-	-	50	36	72	50	36	72
Kondagaon	3	3	-	-	-	29	25	86.2	29	25	86.2
Korba	1	4	40	27	67.5	-	-	-	40	27	67.5
Korea	3	4	-	-	-	40	19	47.5	40	19	47.5
Mahasamund	3	5	50	47	94	-	-	-	50	47	94
Mungeli	3	4	40	30	75	-	-	-	40	30	75
Narayanpur	2	3	30	17	56.7	-	-	-	30	17	56.7
Raigarh	4	8	80	68	85	-	-	-	80	68	85
Raipur	3	4	-	-	-	40	29	72.5	40	29	72.5
Rajnandgaon	5	9	90	78	86.7	-	-	-	90	78	86.7
Sukma	2	3	28	27	96.4	-	-	-	28	27	96.4
Surajpur	2	4	40	40	100	-	-	-	40	40	100
Surguja	4	4	-	-	-	39	30	76.9	39	30	76.9
Bijapur (CH)	2	3	-	-	-	30	19	63.3	30	19	63.3
**Grand Total**	**78**	**118**	**452**	**384**	**85**	**720**	**539**	**74.9**	**1172**	**923**	**78.8**
**Seroconversion at 95% CI**					**CI 95%: (82 to 88)**			**CI 95%: (72 to 78)**			**CI 95%: (76 to 81)**

Table S15: Details of the post-vaccination PPRV antibodies seroconversion in small ruminants in various districts of Madhya Pradesh

			Sheep			Goat			Total		
Name of District	No. of Taluks in the District	No. of Village/ Epi-Units	No. of serum samples screened	No. of samples positive in ELISA	Seroconversion (%)	No. of serum samples screened	No. of samples positive in ELISA	Seroconversion (%)	No. of serum samples screened	No. of samples positive in ELISA	Seroconversion (%)
Agar malwa	2	2	-	-	-	20	10	50	20	10	50
Alirajpur	2	3	-	-	-	30	16	53.3	30	16	53.3
Anuppur	3	3	-	-	-	30	0	0	30	0	0
Ashoknagar	2	3	2	2	100	28	24	85.7	30	26	86.7
Balaghat	3	3	-	-	-	30	25	83.3	30	25	83.3
Barwani	3	3	-	-	-	30	29	96.7	30	29	96.7
Betul	2	3	-	-	-	30	19	63.3	30	19	63.3
Burhanpur	1	1	2	2	100	8	4	50	10	6	60
Chhatarpur	3	3	2	1	50	28	3	10.7	30	4	13.3
Chhindwara	3	5	-	-	-	50	45	90	50	45	90
Damoh	3	3	-	-	-	30	24	80	30	24	80
Datia	2	3	3	0	0	27	0	0	30	0	0
Dewas	2	3	-	-	-	30	23	76.7	30	23	76.7
Dhar	4	4	8	0	0	32	21	65.6	40	21	52.5
East nimar	2	3	1	1	100	29	27	93.1	30	28	93.3
Gwalior	2	3	8	7	87.5	22	17	77.3	30	24	80
Harda	3	3	-	-	-	29	10	34.5	29	10	34.5
Hoshangabad	3	3	-	-	-	30	17	56.7	30	17	56.7
Indore	3	3	-	-	-	30	25	83.3	30	25	83.3
Jabalpur	2	3	4	2	50	25	17	68	29	19	65.5
Jhabua	3	3	-	-	-	30	5	16.7	30	5	16.7
Katni	2	3	1	0	0	29	23	79.3	30	23	76.7
Mandsaur	3	3	2	2	100	28	26	92.9	30	28	93.3
Narsinghpur	2	3	2	1	50	28	15	53.6	30	16	53.3
Panna	2	3	-	-	-	30	11	36.7	30	11	36.7
Ratlam	1	3	-	-	-	30	20	66.7	30	20	66.7
Rewa	3	5	9	8	88.9	41	7	17.1	50	15	30
Satna	3	4	1	1	100	39	20	51.3	40	21	52.5
Sehore	2	3	-	-	-	30	20	66.7	30	20	66.7
Shivpuri	3	3	1	1	100	29	25	86.2	30	26	86.7
Sidhi	3	3	8	8	100	22	22	100	30	30	100
Ujjain	3	3	-	-	-	30	17	56.7	30	17	56.7
Umaria	2	3	-	-	-	30	0	0	30	0	0
Guna	2	3	-	-	-	30	7	23.3	30	7	23.3
Mandla	3	3	-	-	-	30	18	60	30	18	60

**Grand Total**	**87**	**108**	**54**	**36**	**66.7**	**1024**	**592**	**57.8**	**1078**	**628**	**58.3**
**Seroconversion at 95% CI**					**CI 95%: (54 to 79)**			**CI 95%: (55 to 61)**			**CI 95%: (55 to 61)**

Table S16: Details of the post-vaccination PPRV antibodies seroconversion in small ruminants in various districts of Gujarat

Name of District	No. of Taluks in the District	No. of Village/ Epi-Units	No. of serum samples screened	No. of samples positive in ELISA	Seroconversion (%)	No. of serum samples screened	No. of samples positive in ELISA	Seroconversion (%)	No. of serum samples screened	No. of samples positive in ELISA	Seroconversion (%)
			Sheep			Goat			Total		
Ahmadabad	3	3	4	4	100	26	25	96.2	30	29	96.7
Amreli	3	3	15	13	86.7	14	13	92.9	29	26	89.7
Anand	1	3	2	0	0	28	12	42.9	30	12	40
Arvalli	3	4	7	7	100	32	15	46.9	39	22	56.4
Banas Kantha	4	6	10	4	40	50	36	72	60	40	66.7
Bharuch	4	4	-	-	-	38	19	50	38	19	50
Bhavnagar	3	4	14	14	100	24	24	100	38	38	100
Botad	2	3	7	6	85.7	20	18	90	27	24	88.9
Chhotaudepur	2	5	-	-	-	49	21	42.9	49	21	42.9
Devbhumi Dwarka	3	3	20	14	70	10	7	70	30	21	70
Dohad	4	4	-	-	-	38	13	34.2	38	13	34.2
Gandhinagar	2	3	12	4	33.3	17	10	58.8	29	14	48.3
Gir Somnath	1	3	15	15	100	15	15	100	30	30	100
Jamnagar	3	3	23	12	52.2	7	5	71.4	30	17	56.7
Junagadh	2	4	9	4	44.4	31	22	71	40	26	65
Kachchh	3	5	19	7	36.8	31	16	51.6	50	23	46
Kheda	2	4	17	11	64.7	23	14	60.9	40	25	62.5
Mahesana	4	4	2	2	100	38	30	78.9	40	32	80
Mahisagar	3	4	-	-	-	40	9	22.5	40	9	22.5
Morbi	2	3	22	17	77.3	8	3	37.5	30	20	66.7
Narmada	2	4	-	-	-	39	27	69.2	39	27	69.2
Navsari	2	3	1	1	100	29	20	69	30	21	70
Panch Mahals	3	4	-	-	-	39	19	48.7	39	19	48.7
Patan	3	4	15	10	66.7	25	25	100	40	35	87.5
Porbandar	2	3	16	16	100	14	13	92.9	30	29	96.7
Rajkot	3	4	24	13	54.2	16	14	87.5	40	27	67.5
Sabar Kantha	4	5	7	5	71.4	40	26	65	47	31	66
Surat	4	5	1	0	0	49	27	55.1	50	27	54
Surendranagar	2	3	12	1	8.3	16	3	18.8	28	4	14.3
Tapi	2	3	-	-	-	29	24	82.8	29	24	82.8
Vadodara	3	4	-	-	-	40	25	62.5	40	25	62.5
Valsad	2	3	-	-	-	30	30	100	30	30	100
**Grand Total**	**86**	**120**	**274**	**180**	**65.7**	**905**	**580**	**64.1**	**1179**	**760**	**64.5**
**Seroconversion at 95% CI**					**CI 95%: (60 to 71)**			**CI 95%: (61 to 67)**			**CI 95%: (62 to 67)**

Table S17: Details of the post-vaccination PPRV antibodies seroconversion in small ruminants in various districts of Maharashtra

Name of District	No. of Taluks in the State	No. of Village / Epi-Units	Sheep: No. of serum samples screened	Sheep: No. of samples positive in ELISA	Sheep: Seroconversion (%)	Goat: No. of serum samples screened	Goat: No. of samples positive in ELISA	Goat: Seroconversion (%)	Total: No. of serum samples screened	Total: No. of samples positive in ELISA	Total: Seroconversion (%)
Akola	3	3	5	4	80	25	24	96	30	28	93.3
Amravati	4	5	6	6	100	44	33	75	50	39	78
Bhandara	3	3	4	3	75	23	16	69.6	27	19	70.4
Chandrapur	3	4	6	3	50	33	13	39.4	39	16	41
Dhule	2	3	6	6	100	23	19	82.6	29	25	86.2
Gadchiroli	3	4	22	22	100	16	16	100	38	38	100
Gondia	3	3	-	-	-	30	18	60	30	18	60
Jalgaon	4	5	7	7	100	41	41	100	48	48	100
Kolhapur	4	4	3	3	100	34	12	35.3	37	15	40.5
Nagpur	5	5	10	9	90	40	29	72.5	50	38	76
Nandurbar	2	3	-	-	-	24	23	95.8	24	23	95.8
Nashik	2	5	2	2	100	46	29	63	48	31	64.6
Ratnagiri	2	4	-	-	-	17	10	58.8	17	10	58.8
Sangli	3	3	3	2	66.7	23	12	52.2	26	14	53.8
Satara	3	5	4	3	75	44	39	88.6	48	42	87.5
Sindhudurg	2	3	-	-	-	24	13	54.2	24	13	54.2
Solapur	4	4	4	3	75	30	24	80	34	27	79.4
Wardha	2	3	2	2	100	25	25	100	27	27	100
Palghar	3	3	-	-	-	25	25	100	25	25	100
Buldhana	2	3	2	2	100	28	26	92.9	30	28	93.3
Latur	3	3	4	1	25	25	14	56	29	15	51.7
Mumbai	1	1	1	0	0	9	3	33.3	10	3	30
Osmanabad	3	3	2	2	100	27	22	81.5	29	24	82.8
Thane	1	3	-	-	-	23	22	95.7	23	22	95.7
Yavatmal	5	5	2	2	100	48	43	89.6	50	45	90
Hingoli	3	3	4	2	50	22	13	59.1	26	15	57.7
Raigad	4	5	-	-	-	40	35	87.5	40	35	87.5
Washim	3	3	3	3	100	23	13	56.5	26	16	61.5
Aurangabad	8	8	4	2	50	36	22	61.1	40	24	60
Ahmednagar	4	4	13	10	76.9	27	11	40.7	40	21	52.5
Jalna	2	3	3	3	100	27	26	96.3	30	29	96.7

Beed	4	4	3	1	33.3	36	14	38.9	39	15	38.5
Nanded	4	5	5	5	100	41	41	100	46	46	100
**Grand Total**	**104**	**125**	**130**	**108**	**83.1**	**979**	**726**	**74.2**	**1109**	**834**	**75.2**
**Seroconversion at 95% CI**					**CI 95%: (77 to 90)**			**CI 95%: (71 to 77)**			**CI 95%: (73 to 78)**

Table S18: Details of the post-vaccination PPRV antibodies seroconversion in small ruminants in various districts of Rajasthan

Name of District	No. of Taluks in the State	No. of Village/ Epi-Units	No. of serum samples screened	No. of samples positive in ELISA	Seroconversion (%)	No. of serum samples screened	No. of samples positive in ELISA	Seroconversion (%)	No. of serum samples screened	No. of samples positive in ELISA	Seroconversion (%)
			Sheep			Goat			Total		
Ajmer	3	3	-	-	-	30	15	50	30	15	50
Alwar	4	4	-	-	-	40	27	67.5	40	27	67.5
Banswara	3	3	-	-	-	30	22	73.3	30	22	73.3
Baran	3	3	-	-	-	30	22	73.3	30	22	73.3
Bharatpur	3	3	-	-	-	29	17	58.6	29	17	58.6
Bhilwara	4	5	-	-	-	50	4	8	50	4	8
Bundi	2	3	-	-	-	30	16	53.3	30	16	53.3
Churu	3	3	-	-	-	30	23	76.7	30	23	76.7
Dholpur	2	3	-	-	-	30	30	100	30	30	100
Dungarpur	3	3	-	-	-	30	3	10	30	3	10
Hanumangarh	4	5	-	-	-	50	48	96	50	48	96
Jaipur	4	4	-	-	-	40	17	42.5	40	17	42.5
Jaisalmer	2	3	-	-	-	30	5	16.7	30	5	16.7
Jhalawar	3	4	-	-	-	40	33	82.5	40	33	82.5
Karauli	2	3	-	-	-	30	27	90	30	27	90
Kota	2	3	-	-	-	30	27	90	30	27	90
Nagaur	4	5	-	-	-	50	24	48	50	24	48
Rajsamand	3	3	-	-	-	30	14	46.7	30	14	46.7
Tonk	2	3	-	-	-	30	21	70	30	21	70
Jhunjhunu	3	3	10	6	60	20	13	65	30	19	63.3
Bikaner	2	3	-	-	-	30	27	90	30	27	90
Dausa	3	3	-	-	-	30	18	60	30	18	60
Ganganagar	4	4	-	-	-	40	36	90	40	36	90
Jodhpur	5	5	-	-	-	41	32	78	41	32	78
Chittorgarh	2	3	-	-	-	30	30	100	30	30	100
Udaipur	3	4	-	-	-	40	23	57.5	40	23	57.5
Pali	3	3	-	-	-	30	22	73.3	30	22	73.3
**Grand Total**	**81**	**94**	**10**	**6**	**60**	**920**	**596**	**64.8**	**930**	**602**	**64.7**
**Seroconversion at 95% CI**					**CI 95%: (30 to 90)**			**CI 95%: (62 to 68)**			**CI 95%: (62 to 68)**

Table S19: Details of the post-vaccination PPRV antibodies seroconversion in small ruminants in various districts of Haryana

Name of District	No. of Taluks in the State	No. of Village/ Epi-Units	No. of serum samples screened	No. of samples positive in ELISA	Seroconversion (%)	No. of serum samples screened	No. of samples positive in ELISA	Seroconversion (%)	No. of serum samples screened	No. of samples positive in ELISA	Seroconversion (%)
			Sheep			Goat			Total		
Ambala	3	6	51	40	78.4	9	8	88.9	60	48	80
Bhiwani	3	5	32	22	68.8	18	16	88.9	50	38	76
Charki dadri	1	3	16	10	62.5	14	9	64.3	30	19	63.3
Faridabad	2	3	15	15	100	15	12	80	30	27	90
Fatehabad	2	5	40	33	82.5	10	9	90	50	42	84
Gurugram	3	5	21	18	85.7	29	28	96.6	50	46	92
Hisar	3	5	22	14	63.6	28	22	78.6	50	36	72
Jhajjar	3	5	36	17	47.2	14	7	50	50	24	48
Jind	2	5	36	18	50	14	9	64.3	50	27	54
Kaithal	2	5	37	12	32.4	12	10	83.3	49	22	44.9
Karnal	5	8	56	44	78.6	23	15	65.2	79	59	74.7
Kurukshetra	3	7	59	31	52.5	11	10	90.9	70	41	58.6
Mahendragarh	2	5	15	15	100	35	35	100	50	50	100
Mewat	2	5	9	9	100	41	38	92.7	50	47	94
Palwal	3	5	31	23	74.2	18	13	72.2	49	36	73.5
Panchkula	2	3	18	9	50	12	5	41.7	30	14	46.7
Panipat	2	3	29	22	75.9	1	1	100	30	23	76.7
Rewari	1	7	17	13	76.5	53	47	88.7	70	60	85.7
Rohtak	2	3	18	14	77.8	12	9	75	30	23	76.7
Sirsa	3	5	20	15	75	30	24	80	50	39	78
Sonipat	2	5	33	20	60.6	17	9	52.9	50	29	58
Yamunanagar	3	9	70	52	74.3	19	11	57.9	89	63	70.8
**Grand Total**	**54**	**112**	**681**	**466**	**68.4**	**435**	**347**	**79.8**	**1116**	**813**	**72.8**
**Seroconversion at 95% CI**					**CI 95%: (65 to 72)**			**CI 95%: (76 to 84)**			**CI 95%: (70 to 75)**

Table S20: Details of the post-vaccination PPRV antibodies seroconversion in small ruminants in various districts of Himachal Pradesh

Name of District	No. of Taluks in the State	No. of Village/ Epi-Units	No. of serum samples screened	No. of samples positive in ELISA	Seroconversion (%)	No. of serum samples screened	No. of samples positive in ELISA	Seroconversion (%)	No. of serum samples screened	No. of samples positive in ELISA	Seroconversion (%)
			Sheep			Goat			Total		
Bilaspur	8	9	-	-	-	39	26	66.7	39	26	66.7
Hamirpur	4	7	29	6	20.7	25	6	24	54	12	22.2
Chamba	4	7	36	24	66.7	33	18	54.5	69	42	60.9
Kangra	9	23	70	28	40	142	91	64.1	212	119	56.1
Kinnaur	3	3	21	17	81	9	6	66.7	30	23	76.7
Kullu	2	3	13	5	38.5	16	16	100	29	21	72.4
Mandi	10	18	68	13	19.1	100	49	49	168	62	36.9
Shimla	4	14	98	69	70.4	36	15	41.7	134	84	62.7
Solan	6	9	-	-	-	81	51	63	81	51	63
**Grand Total**	**50**	**93**	**335**	**162**	**48.4**	**481**	**278**	**57.8**	**816**	**440**	**53.9**
**Seroconversion at 95% CI**					**CI 95%: (43 to 54)**			**CI 95%: (53 to 62)**			**CI 95%: (51 to 57)**

Table S21: Details of the post-vaccination PPRV antibodies Seroconversion in small ruminants in various districts of Punjab

Name of District	No. of Taluks in the District	No. of Village/ Epi-Units	No. of serum samples screened	No. of samples positive in ELISA	Seroconversion (%)	No. of serum samples screened	No. of samples positive in ELISA	Seroconversion (%)	No. of serum samples screened	No. of samples positive in ELISA	Seroconversion (%)
			Sheep			Goat			Total		
Amritsar	4	5	5	5	100	45	34	75.6	50	39	78
Barnala	1	3	-	-	-	30	2	6.7	30	2	6.7
Bathinda	3	5	2	2	100	48	39	81.3	50	41	82
Faridkot	3	5	-	-	-	50	43	86	50	43	86
Fatehgarh Sahib	2	4	-	-	-	40	33	82.5	40	33	82.5
Fazilka	2	3	16	12	75	14	8	57.1	30	20	66.7
Firozepur	2	5	-	-	-	50	43	86	50	43	86
Gurdaspur	2	5	10	3	30	34	15	44.1	44	18	40.9
Hoshiarpur	3	10	16	16	100	75	46	61.3	91	62	68.1
Jalandhar	3	5	9	4	44.4	40	15	37.5	49	19	38.8
Kapurthala	3	5	-	-	-	50	42	84	50	42	84
Ludhiana	2	5	-	-	-	50	43	86	50	43	86
Mansa	2	3	5	3	60	25	10	40	30	13	43.3
Moga	4	5	-	-	-	50	31	62	50	31	62
Muktsar	4	5	-	-	-	50	47	94	50	47	94
Nawanshahr (SBS Nagar)	4	5	-	-	-	50	36	72	50	36	72
Pathankot	2	4	10	10	100	30	21	70	40	31	77.5
Patiala	2	8	60	22	36.7	16	6	37.5	76	28	36.8
Rupnagar	4	5	-	-	-	50	35	70	50	35	70
S.A.S Nagar	3	5	20	20	100	29	11	37.9	49	31	63.3
Sangrur	2	5	-	-	-	50	35	70	50	35	70
Taran	2	2	-	-	-	20	19	95	20	19	95
**Grand Total**	**59**	**107**	**153**	**97**	**63.4**	**896**	**614**	**68.5**	**1049**	**711**	**67.8**
**Seroconversion at 95% CI**					**CI 95%: (56 to 71)**			**CI 95%: (65 to 72)**			**CI 95%: (65 to 71)**

Table S22: Details of the post-vaccination PPRV antibodies seroconversion in small ruminants in various districts of Uttar Pradesh

Name of District	No. of Taluks in the State	No. of Village/ Epi-Units	No. of serum samples screened	No. of samples positive in ELISA	Seroconversion (%)	No. of serum samples screened	No. of samples positive in ELISA	Seroconversion (%)	No. of serum samples screened	No. of samples positive in ELISA	Seroconversion (%)
			Sheep			Goat			Total		
Mirzapur	3	3	1	0	0	29	22	75.9	30	22	73.3
Basti	1	3	1	1	100	29	29	100	30	30	100
Bijnor	2	3	1	1	100	29	24	82.8	30	25	83.3
Etawah	1	1	-	-	-	10	0	0	10	0	0
Farrukhabad	1	3	-	-	-	30	27	90	30	27	90
Gautam Buddha Nagar	1	1	-	-	-	10	7	70	10	7	70
Ghazipur	3	3	-	-	-	30	22	73.3	30	22	73.3
Hathras	1	1	-	-	-	10	10	100	10	10	100
Kannauj	1	1	1	1	100	9	8	88.9	10	9	90
Kanpur Dehat	2	3	2	2	100	28	21	75	30	23	76.7
Kanpur Nagar	3	3	1	0	0	29	24	82.8	30	24	80
Kasganj	1	1	-	-	-	10	8	80	10	8	80
Kaushambi	1	1	1	1	100	9	6	66.7	10	7	70
Lalitpur	1	1	-	-	-	10	10	100	10	10	100
Mainpuri	1	1	1	1	100	9	9	100	10	10	100
Mathura	1	1	1	1	100	9	5	55.6	10	6	60
Muzaffarnagar	1	1	-	-	-	10	9	90	10	9	90
Pilibhit	1	2	1	0	0	19	5	26.3	20	5	25
Rae Bareli	2	2	2	0	0	18	4	22.2	20	4	20
Rampur	3	3	-	-	-	30	26	86.7	30	26	86.7
Sant Kabeer Nagar	3	3	2	2	100	28	28	100	30	30	100
Sant Ravidas Nagar	1	3	1	1	100	29	21	72.4	30	22	73.3
Shravasti	1	1	-	-	-	10	10	100	10	10	100
Siddharth Nagar	3	3	2	0	0	28	3	10.7	30	3	10
Sultanpur	2	3	1	1	100	29	17	58.6	30	18	60
Unnao	3	3	3	2	66.7	27	11	40.7	30	13	43.3
Agra	1	2	1	1	100	18	14	77.8	19	15	78.9
Aligarh	3	3	2	2	100	28	21	75	30	23	76.7
Ambedkar Nagar	2	3	1	0	0	29	0	0	30	0	0
Auraiya	1	1	-	-	-	10	10	100	10	10	100
Azamgarh	4	4	2	1	50	38	24	63.2	40	25	62.5
Baghpat	1	1	1	1	100	9	7	77.8	10	8	80
Bahraich	2	2	1	1	100	19	19	100	20	20	100
Ballia	2	2	1	0	0	19	6	31.6	20	6	30
Barabanki	1	1	1	1	100	9	5	55.6	10	6	60

Bareilly	3	3	2	1	50	27	9	33.3	29	10	34.5
Bulandshahr	3	3	2	2	100	28	25	89.3	30	27	90
Chandauli	2	3	1	0	0	22	13	59.1	23	13	56.5
Chitrakoot	1	1	-	-	-	10	0	0	10	0	0
Deoria	2	3	2	0	0	28	0	0	30	0	0
Fatehpur	2	3	1	0	0	29	9	31	30	9	30
Firozabad	1	1	1	1	100	9	9	100	10	10	100
Ghaziabad	1	1	1	1	100	9	9	100	10	10	100
Gorakhpur	4	4	3	3	100	36	33	91.7	39	36	92.3
Hamirpur	4	7	1	1	100	9	9	100	10	10	100
Hapur	1	1	-	-	-	10	10	100	10	10	100
Hardoi	3	3	2	1	50	28	21	75	30	22	73.3
Jalaun	3	3	3	3	100	27	19	70.4	30	22	73.3
Jaunpur	3	4	1	1	100	39	31	79.5	40	32	80
Jhansi	2	3	2	0	0	27	6	22.2	29	6	20.7
Mau	2	3	2	1	50	28	5	17.9	30	6	20
Meerut	1	1	-	-	-	10	10	100	10	10	100
Moradabad	2	3	2	0	0	28	9	32.1	30	9	30
Sambhal	2	3	2	1	50	28	19	67.9	30	20	66.7
Sitapur	3	3	2	2	100	28	26	92.9	30	28	93.3
Sonbhadra	1	2	2	0	0	15	0	0	17	0	0
**Grand Total**	**107**	**131**	**64**	**39**	**60.9**	**1172**	**744**	**63.5**	**1236**	**783**	**63.3**
**Seroconversion at 95% CI**					**CI 95%: (49 to 73)**			**CI 95%: (61 to 66)**			**CI 95%: (61 to 66)**

Table S23: Details of the post-vaccination PPRV antibodies seroconversion in small ruminants in various districts of Assam

Name of District	No. of Taluks in the State	No. of Village/ Epi-Units	No. of serum samples screened	No. of samples positive in ELISA	Seroconversion (%)	No. of serum samples screened	No. of samples positive in ELISA	Seroconversion (%)	No. of serum samples screened	No. of samples positive in ELISA	Seroconversion (%)
			Sheep			Goat			Total		
Barpeta	4	4	15	7	46.7	25	19	76	40	26	65
Biswanath	3	4	-	-	-	40	24	60	40	24	60
Bongaigaon	3	3	3	-	-	26	21	80.8	29	21	72.4
Cachar	2	5	9	5	55.6	41	20	48.8	50	25	50
Chirang	3	3	-	-	-	30	17	56.7	30	17	56.7
Darrang	2	3	-	-	-	30	26	86.7	30	26	86.7
Dhemaji	4	9	-	-	-	89	20	22.5	89	20	22.5
Dhubri	2	3	11	5	45.5	19	12	63.2	30	17	56.7
Dibrugarh	4	5	-	-	-	49	30	61.2	49	30	61.2
Dima Hasao	3	3	-	-	-	29	24	82.8	29	24	82.8
Goalpara	3	4	8	4	50	31	21	67.7	39	25	64.1
Golaghat	5	5	-	-	-	49	44	89.8	49	44	89.8
Hailakandi	2	2	-	-	-	20	15	75	20	15	75
Hojai	2	3	-	-	-	30	17	56.7	30	17	56.7
Jorhat	1	3	-	-	-	30	27	90	30	27	90
Kamrup	4	5	-	-	-	50	39	78	50	39	78
Kamrup (M)	3	3	-	-	-	30	14	46.7	30	14	46.7
Karbi Anglong	3	9	-	-	-	84	67	79.8	84	67	79.8
Kokrajhar	3	4	20	0	0	20	19	95	40	19	47.5
Lakhimpur	3	5	-	-	-	49	41	83.7	49	41	83.7
Majuli	1	1	-	-	-	10	9	90	10	9	90
Nagaon	3	4	-	-	-	40	30	75	40	30	75
Nalbari	3	3	-	-	-	30	21	70	30	21	70
Sibsagar	2	3	-	-	-	30	27	90	30	27	90
Sonitpur	3	4	-	-	-	39	28	71.8	39	28	71.8
Soraideu	2	3	10	8	80	19	17	89.5	29	25	86.2
South Salmara	1	1	-	-	-	10	9	90	10	9	90
Tinsukia	2	5	-	-	-	50	45	90	50	45	90
Udalguri	2	4	-	-	-	40	36	90	40	36	90
West Karbi Anglong	1	3	-	-	-	30	30	100	30	30	100
**Grand Total**	**79**	**116**	**76**	**29**	**38.2**	**1069**	**769**	**71.9**	**1145**	**798**	**69.7**
**Seroconversion at 95% CI**					**CI 95%: (27 to 49)**			**CI 95%: (69 to 75)**			**CI 95%: (67 to 72)**

Table S24: Details of the post-vaccination PPRV antibodies seroconversion in small ruminants in various districts of Manipur

Name of District	No. of Taluks in the State	No. of Village/ Epi-Units	No. of serum samples screened	No. of samples positive in ELISA	Seroconversion (%)	No. of serum samples screened	No. of samples positive in ELISA	Seroconversion (%)	No. of serum samples screened	No. of samples positive in ELISA	Seroconversion (%)
			Sheep			Goat			Total		
Chandel	4	10	2	0	0	86	48	55.8	88	48	54.5
Senapati	6	24	-	-	-	211	121	57.3	211	121	57.3
Bishnupur	2	4	-	-	-	36	21	58.3	36	21	58.3
Churachandpur	5	19	3	1	33.3	168	103	61.3	171	104	60.8
Imphal East	4	13	-	-	-	117	79	67.5	117	79	67.5
Imphal West	6	9	-	-	-	80	36	45	80	36	45
Tamenglong	3	5	-	-	-	45	27	60	45	27	60
Thoubal	4	8	-	-	-	72	32	44.4	72	32	44.4
Ukhrul	4	5	-	-	-	45	32	71.1	45	32	71.1
**Grand Total**	**38**	**97**	**5**	**1**	**20**	**860**	**499**	**58**	**865**	**500**	**57.8**
**Seroconversion at 95% CI**					**CI 95%: (-15 to 55)**			**CI 95%: (55 to 61)**			**CI 95%: (55 to 61)**

Table S25: Details of the post-vaccination PPRV antibodies seroconversion in small ruminants in various districts of Meghalaya

Name of District	No. of Taluks in the State	No. of Village/ Epi-Units	No. of serum samples screened	No. of samples positive in ELISA	Seroconversion (%)	No. of serum samples screened	No. of samples positive in ELISA	Seroconversion (%)	No. of serum samples screened	No. of samples positive in ELISA	Seroconversion (%)
			Sheep			Goat			Total		
East Garo Hills	2	8	-	-	-	73	34	46.6	73	34	46.6
East Jaintia Hills	1	4	-	-	-	38	23	60.5	38	23	60.5
East Khasi Hills	3	3	-	-	-	28	15	53.6	28	15	53.6
North Garo Hills	3	9	-	-	-	81	5	6.2	81	5	6.2
Ri Bhoi	3	5	-	-	-	46	40	87	46	40	87
South Garo Hills	4	13	-	-	-	120	13	10.8	120	13	10.8
South West Garo Hills	2	8	-	-	-	73	66	90.4	73	66	90.4
South west Khasi Hills	1	2	-	-	-	19	19	100	19	19	100
West Garo Hills	4	14	-	-	-	124	68	54.8	124	68	54.8
West Jaintia Hills	2	5	-	-	-	45	35	77.8	45	35	77.8
West Khasi Hills	1	5	-	-	-	47	37	78.7	47	37	78.7
Eastren West Khasi Hills	1	3	-	-	-	29	29	100	29	29	100
**Grand Total**	**27**	**79**	-	-	-	**723**	**384**	**53.1**	**723**	**384**	**53.1**
**Seroconversion at 95% CI**								**CI 95%: (49 to 57)**			**CI 95%: (49 to 57)**

Table S26: Details of the post-vaccination PPRV antibodies seroconversion in small ruminants in various districts of Mizoram

Name of District	No. of Taluks in the District	No. of Village/ Epi-Units	No. of serum samples screened	No. of samples positive in ELISA	Seroconversion (%)	No. of serum samples screened	No. of samples positive in ELISA	Seroconversion (%)	No. of serum samples screened	No. of samples positive in ELISA	Seroconversion (%)
			Sheep			Goat			Total		
Lawngtlai	3	22	25	0	0	190	190	100	215	190	88.4
Mamit	3	10	35	0	0	85	85	100	120	85	70.8
Kolasib	3	6	16	3	18.8	53	50	94.3	69	53	76.8
Lunglei	4	22	60	0	0	193	193	100	253	193	76.3
Siaha	2	12	25	0	0	104	104	100	129	104	80.6
**Grand Total**	**15**	**72**	**161**	**3**	**1.9**	**625**	**622**	**99.5**	**786**	**625**	**79.5**
**Seroconversion at 95% CI**					**CI 95%: (0 to 4)**			**CI 95%: (99 to 100)**			**CI 95%: (77 to 82)**

Table S27: Details of the post-vaccination PPRV antibodies seroconversion in small ruminants in various districts of Sikkim

Name of District	No. of Taluks in the State	No. of Village/ Epi-Units	No. of serum samples screened	No. of samples positive in ELISA	Seroconversion (%)	No. of serum samples screened	No. of samples positive in ELISA	Seroconversion (%)	No. of serum samples screened	No. of samples positive in ELISA	Seroconversion (%)
			**Sheep**			**Goat**			**Total**		
East District	5	26	-	-	-	254	157	61.8	254	157	61.8
North District	2	3	-	-	-	27	17	63	27	17	63
West District	4	29	4	2	50	284	189	66.5	288	191	66.3
South District	4	28	-	-	-	272	137	50.4	272	137	50.4
**Grand Total**	**15**	**86**	**4**	**2**	**50**	**837**	**500**	**59.7**	**841**	**502**	**59.7**
**Seroconversion at 95% CI**					**CI 95%: (1 to 99)**			**CI 95%: (56 to 63)**			**CI 95%: (56 to 63)**

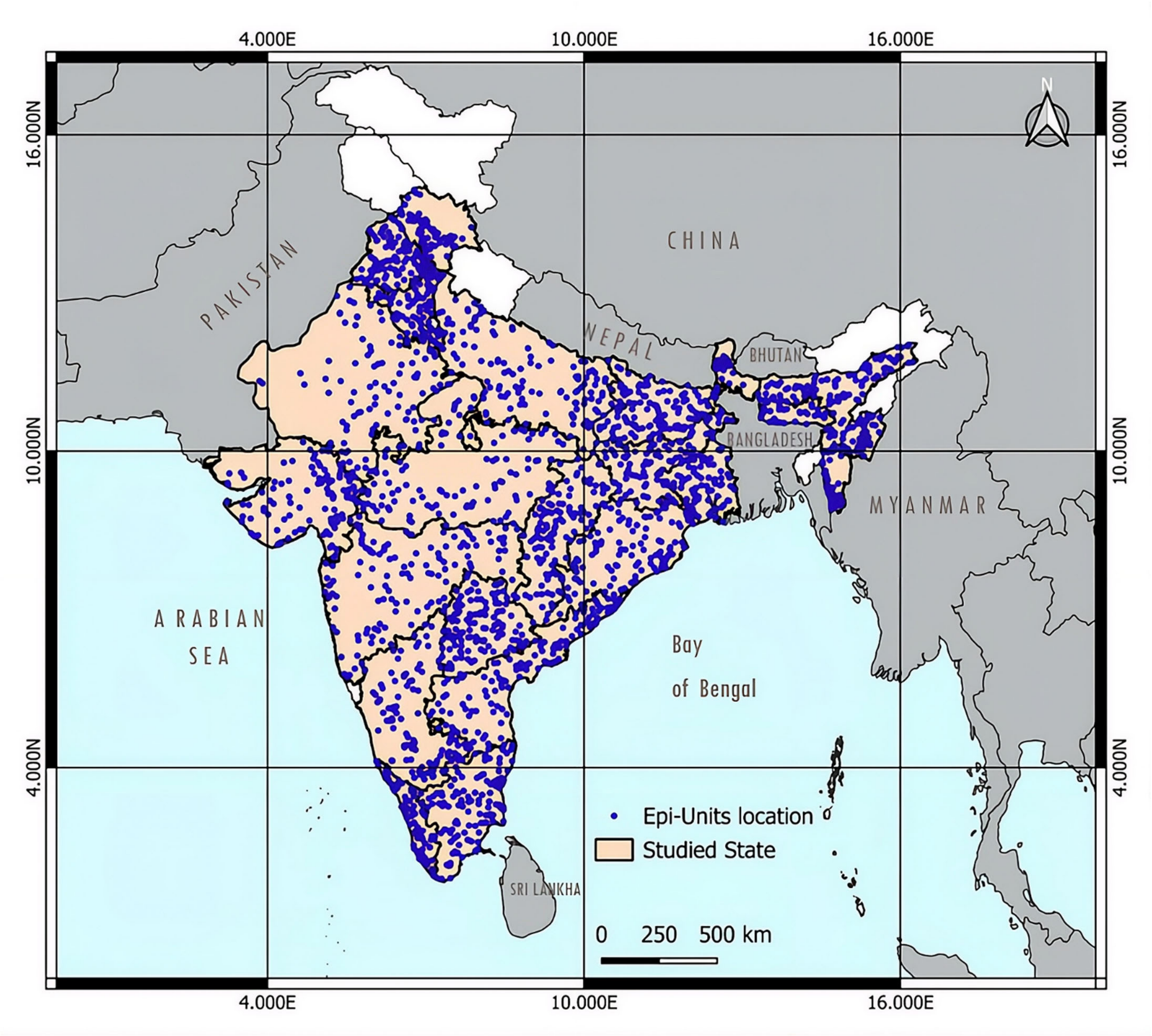

Figure S1: Locations of surveyed epidemiological units-epi-units (villages) across 24 Indian states and union territories are depicted as dot in GIS-based maps of India

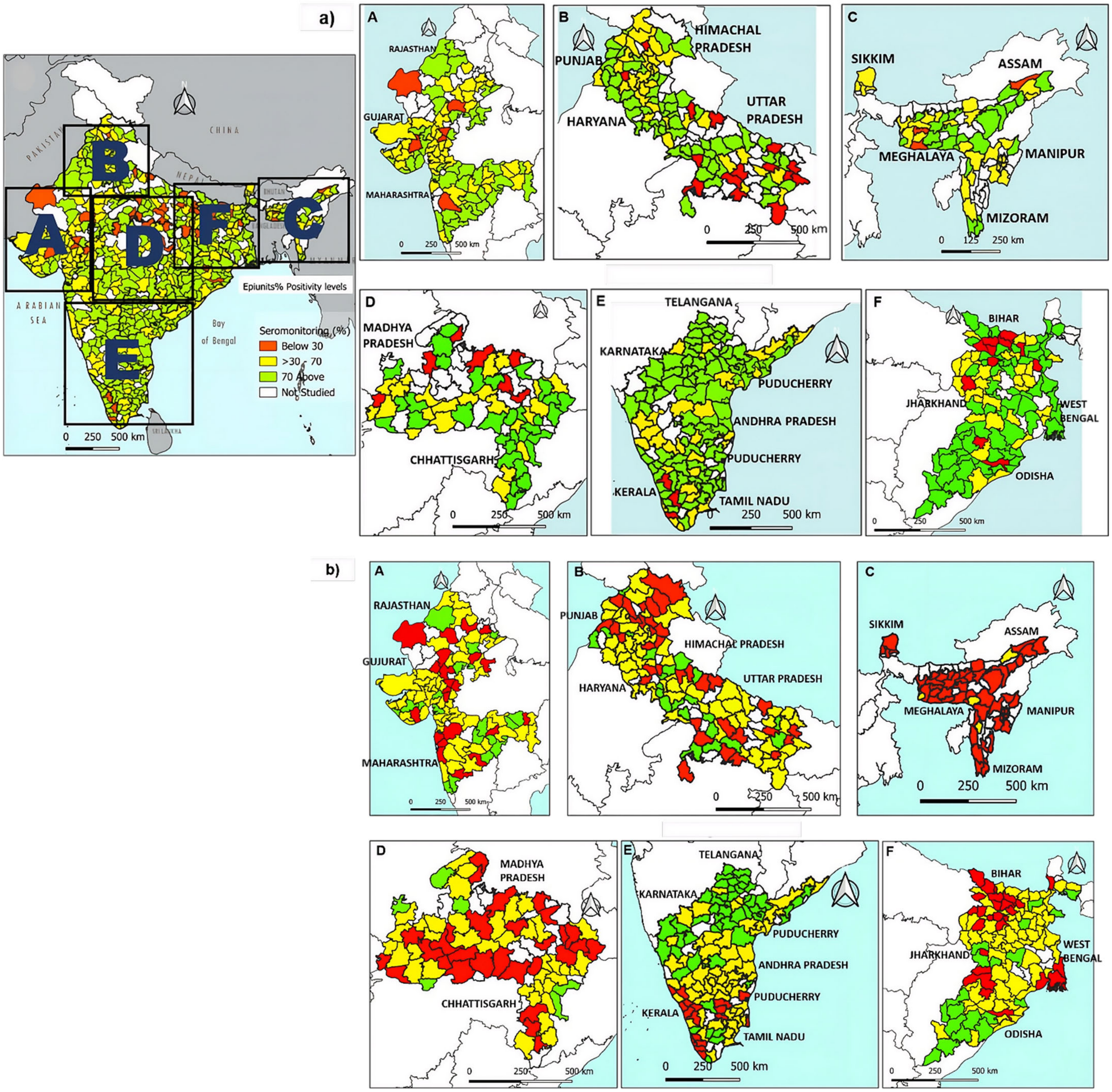

Figure S2: District-wise distribution of PPR virus immune response antibody in small ruminants across studied states, covering multiple epidemiological units after the first annual mass vaccination.

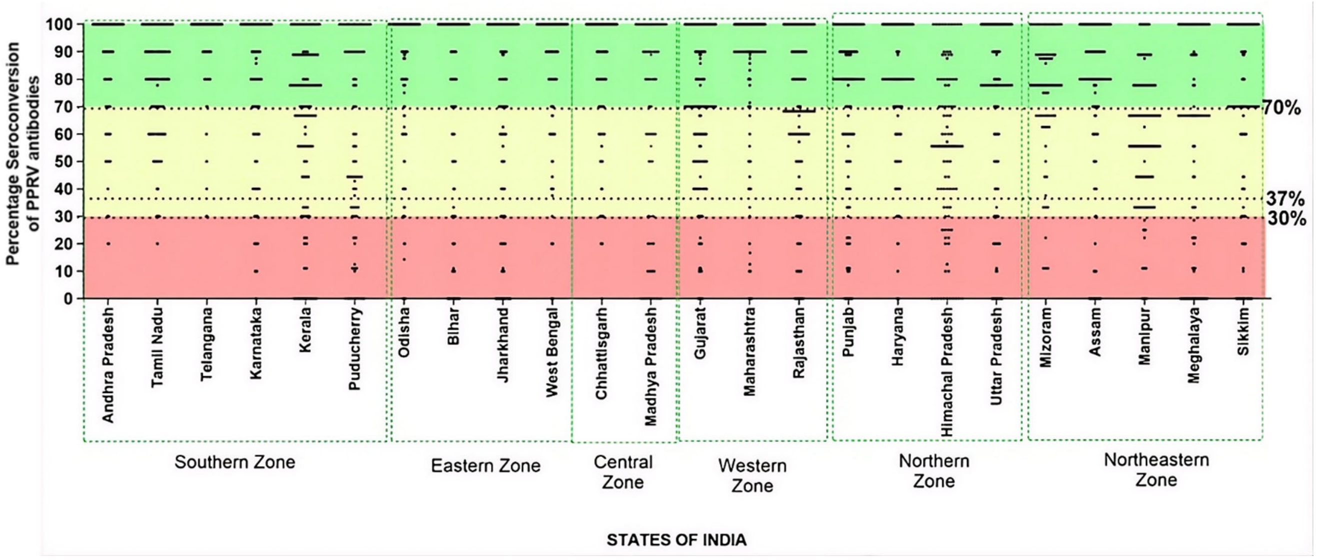

Figure S3: Distribution of epi-units based on post-vaccination PPRV antibody immune response across studied states in India. These sero-monitoring results for PPRV antibodies in small ruminants across surveyed states show the immunity status at the state level, highlighting the percentage prevalence of antibodies in small ruminants aged 6 −12 months after first annual mass vaccination.
